# Recent Advances in the Degradability and Applications of Tissue Adhesives Based on Biodegradable Polymers

**DOI:** 10.3390/ijms25105249

**Published:** 2024-05-11

**Authors:** Shuzhuang Zhu, Wenguang Dou, Xiaojun Zeng, Xingchao Chen, Yonglin Gao, Hongliang Liu, Sidi Li

**Affiliations:** 1 College of Chemistry and Chemical Engineering, Yantai University, Yantai 264005, China; 2 College of Life Sciences, Yantai University, Yantai 264005, China

**Keywords:** biodegradable polymers, tissue adhesives, natural polymers, synthetic polymers, wound healing, tissue engineering

## Abstract

In clinical practice, tissue adhesives have emerged as an alternative tool for wound treatments due to their advantages in ease of use, rapid application, less pain, and minimal tissue damage. Since most tissue adhesives are designed for internal use or wound treatments, the biodegradation of adhesives is important. To endow tissue adhesives with biodegradability, in the past few decades, various biodegradable polymers, either natural polymers (such as chitosan, hyaluronic acid, gelatin, chondroitin sulfate, starch, sodium alginate, glucans, pectin, functional proteins, and peptides) or synthetic polymers (such as poly(lactic acid), polyurethanes, polycaprolactone, and poly(lactic-co-glycolic acid)), have been utilized to develop novel biodegradable tissue adhesives. Incorporated biodegradable polymers are degraded in vivo with time under specific conditions, leading to the destruction of the structure and the further degradation of tissue adhesives. In this review, we first summarize the strategies of utilizing biodegradable polymers to develop tissue adhesives. Furthermore, we provide a symmetric overview of the biodegradable polymers used for tissue adhesives, with a specific focus on the degradability and applications of these tissue adhesives. Additionally, the challenges and perspectives of biodegradable polymer-based tissue adhesives are discussed. We expect that this review can provide new inspirations for the design of novel biodegradable tissue adhesives for biomedical applications.

## 1. Introduction

**Figure 1:**
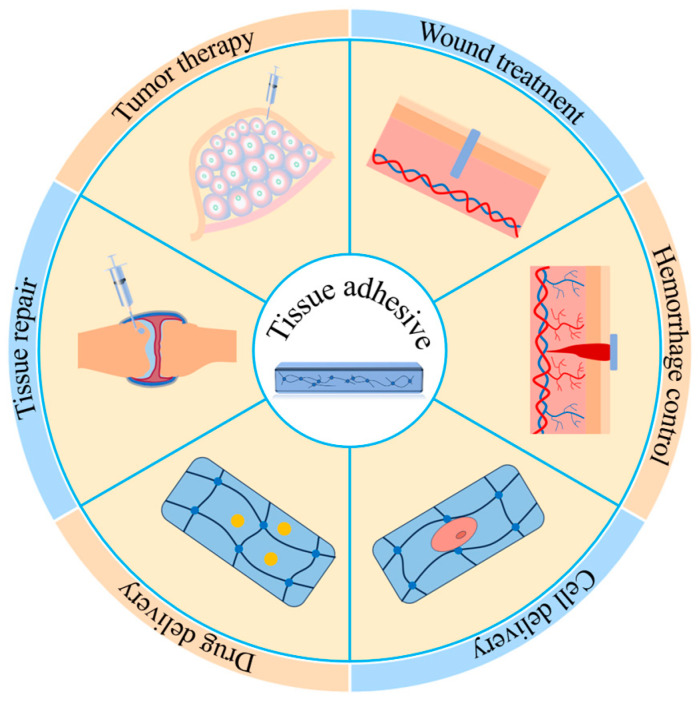
Applications of tissue adhesives. Tissue adhesives have been widely used in various fields.

Millions of people worldwide suffer from accidental or surgical wounds each year [1,2]. Clinically, surgical sutures, staples, clips, and skin closure strips are commonly used tools to close open wounds [3,4,5]. Surgical sutures, staples, and clips close open wounds by physically interlocking the injured tissue. Skin closure strips are like adhesive tapes and can be used to approximate wound edges by adhering wound-surrounding tissue. Although the closure procedures through these tools are considered the gold standard for wound closure [6], each of these methods has its own drawbacks. For example, as the first choice of wound closure, suturing requires time-consuming, professional operations of medical workers and often causes secondary damage and microbial infections of the treated tissues [7,8]. In addition, the pain produced during the suturing process also results in the discomfort of patients [9]. Staples and clips close the wounds rapidly by a simple “click” operation. However, scars are easily produced, and patients usually suffer from more pain during the removal of the tools after wound healing [4]. Skin closure strips, an alternative wound closure tool, show advantages in easy operation and less scars, but they usually show low tensile strength and are not suitable for wounds in wet and hairy environments [10]. In the last few decades, tissue adhesives have emerged as an attractive tool in wound treatments. Different from traditional wound closure tools, the working principle of tissue adhesives in closing open wounds is bonding the injured tissues. To achieve this, adhesives are required to adhere to the tissue surface and be able to hold the two sides of injured tissues together. Therefore, generally, tissue adhesives should show both strong interactions with tissues and cohesive strength. Tissue adhesives show many advantages such as ease of use, rapid application, less pain, and minimal tissue damage [11,12,13,14]. Currently, tissue adhesives have been widely applied in a variety of fields, mainly including acute or infective wound treatments [15,16], hemorrhage control [17,18], cell delivery [19], drug delivery [20], tissue repair [21,22], and tumor therapy [23,24] (Figure 1). The rapid development of tissue adhesives provides a new and alternative choice for medical workers in wound or disease treatment. 

**Figure 2:**
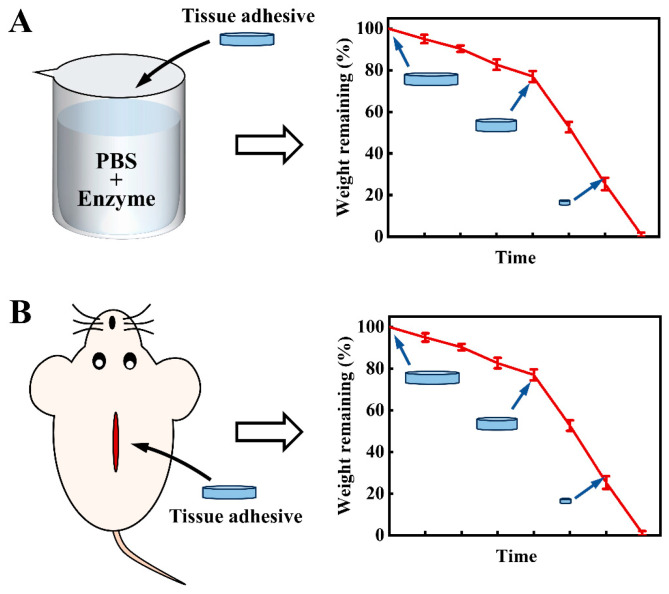
The common methods used for the measurement of the degradation of tissue adhesives in vitro (**A**) and in vivo (**B**).

To better treat wounds and accelerate wound healing, tissue adhesives are usually required to exhibit multiple properties. Apart from the basic tissue adhesiveness, generally, tissue adhesives should possess biocompatibility [12,25,26], degradability [27,28], mechanical compliance with underlying tissues [1,29], an appropriate swelling ratio [2,30,31], storage stability [1,32], etc. In addition, for some applications, some specific features are also required. For example, antibacterial properties are necessary for the treatment of infective wounds [33]. Self-healing properties are often required for the stable closure of dynamic wounds [34]. The on-demand degradation or dissolution property is important for the care of burn wounds [35]. Among these properties, the degradation of tissue adhesives is often necessary, especially for those used in vivo [2,27,28,36]. The degradation of tissue adhesives can avoid the complicated and even injurious adhesive-removal process, thereby benefiting wound healing. The degradability of tissue adhesives can be measured in vitro and in vivo (Figure 2). In the in vitro degradation test, the tissue adhesives are put into a solution that mimics the environment of a body, and in the in vivo test, the tissue adhesives are directly implanted into the bodies. The changes in size and weight of the tissue adhesives with time indicate the degradation results of the tissue adhesives. 

Since the healing time of tissues is usually different due to the differences in tissue types, wound types, and sizes, the ideal degradation time for tissue adhesives is commonly different. Thus, it is hard to directly point out the ideal, specific degradation time for each type of application. In an ideal circumstance, internally applied tissue adhesives should be degraded with an appropriate speed that matches the wound-healing process [2,37]. The slow degradation of tissue adhesives causes the delayed healing of wounds [11], and fast degradation makes an adhesive lose its structure and functions before wound healing [4]. Therefore, the design of appropriate degradable tissue adhesives is of vital importance. 

The most common way to develop degradable tissue adhesives is to utilize biodegradable polymers, either natural or synthetic ones, as the basic building units [38,39]. Currently, many biodegradable polymer-based tissue adhesives have been commercialized (Table 1). The degradation of biodegradable polymers under specific conditions can lead to the destruction of the network structure and the final degradation of the tissue adhesives. Based on this idea, a variety of degradable tissue adhesives have been developed in the past few decades and have been widely used in various fields by virtue of their degradability as well as other fascinating functions [40,41,42]. This review first summarizes the main strategies for the development of tissue adhesives by utilizing biodegradable polymers. Furthermore, an overview of natural or synthetic biodegradable polymer-based tissue adhesives is provided, with a specific focus on the degradability and applications of these tissue adhesives. Since a single component is usually difficult to form an adhesive and another polymer is often necessary, the tissue adhesives formed by both natural and synthetic polymers are included either in the natural or synthetic polymer parts and are not listed separately. The challenges and perspectives of biodegradable polymer-based tissue adhesives are also discussed at the end of this review.

## 2. General Design Strategies for Biodegradable Polymer-Based Tissue Adhesives

**Figure 3:**
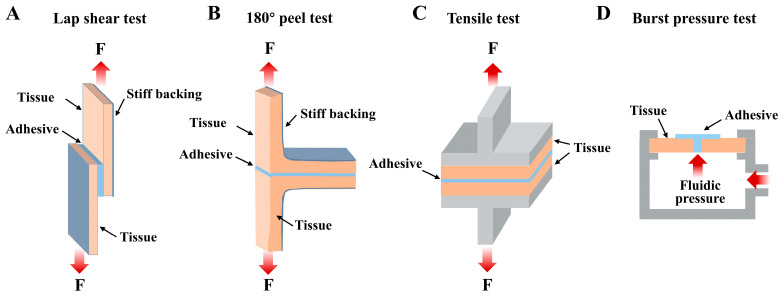
The common methods used for the measurement of the adhesive performance of tissue adhesives. (**A**) Lap shear test; (**B**) 180° peel test; (**C**) tensile test; (**D**) burst pressure test.

Generally, tissue adhesives are stored in a liquid state, and upon application at the target tissue, the liquid tissue adhesives solidify through multiple types of crosslinking, thereby bonding the biological tissues [17]. Adhesive performance is essential for tissue adhesives. Many methods have been reported for measuring the adhesive performance of a tissue adhesive such as the lap shear test, the 180° peel test, the tensile test, and the burst pressure test (Figure 3). 

**Figure 4:**
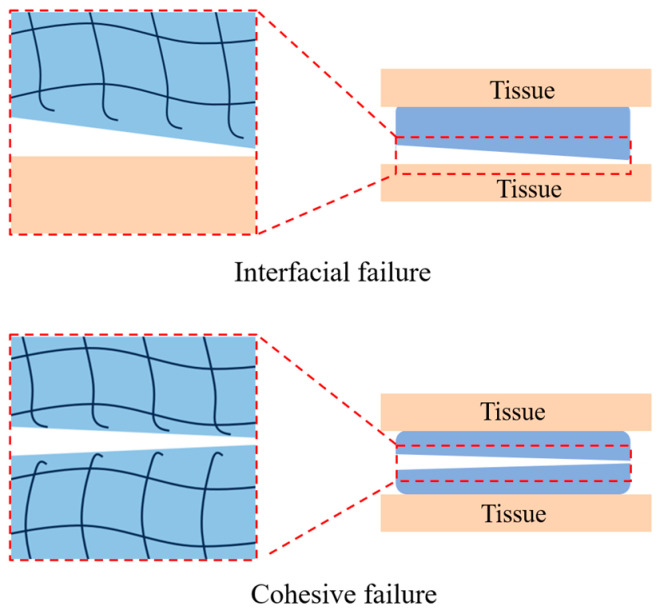
Schematic representation of interfacial failure and cohesive failure.

To achieve stable tissue adhesion, both the interfacial adhesion and cohesive strength of the tissue adhesives are important and should be considered in the design of tissue adhesives [43,44]. If the interfacial adhesion capacity of a tissue adhesive is poor, the bonded sample is prone to undergo interfacial failure [45]. And if the cohesive strength is low, cohesive failure will occur (Figure 4) [46]. Over the past few decades, various strategies for improving the interfacial adhesion or cohesive strength of biodegradable polymer-based tissue adhesives were proposed. Commonly, interfacial adhesion can be improved by enhancing the interactions between the adhesives and the biological tissues [2], and cohesive strength can be improved by solidification that is physically or chemically crosslinking induced [47]. 

### 2.1. Strategies for Improving Interfacial Adhesion

**Figure 5:**
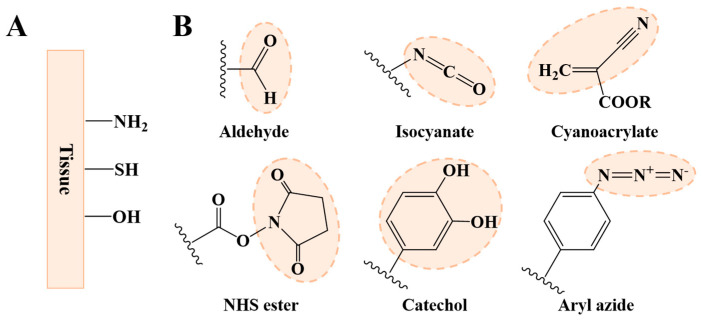
Interfacial adhesion. (**A**) Main groups in biological tissues for the design of tissue adhesives. (**B**) The functional groups used for enhancing interfacial adhesion.

There are many functional groups originating from amino acid residues in biological tissues that can be used for tissue adhesion (Figure 5A). The most commonly used functional groups are primary amino, hydroxy, and sulfydryl groups [48]. These functional groups can form covalent linking with some specific groups, such as the aldehyde group, *N*-hydroxysuccinimide (NHS) esters, isocyanates, catechol, cyanoacrylate, aryl azide, etc. (Figure 5B) [49]. However, these groups are absent in common biodegradable polymers. Therefore, introducing these groups through some modifications is often necessary for improving interfacial adhesion. For example, Zhou and coworkers introduced the catechol group into hyaluronic acid through a Schiff-base reaction between the amino groups of dopamine and the aldehyde group of aldehyde-modified sodium hyaluronate. After oxidized crosslinking, they found the maximal adhesive strength of the catechol-modified hyaluronic acid adhesive reached about 90 kPa, which is much higher than commercial fibrin glue [50]. 

Apart from forming covalent linking, some noncovalent interactions were also employed to improve the interfacial adhesion. These noncovalent interactions include physical mechanical interlocking, hydrogen bonding (H-bonding), electrostatic interactions, hydrophobic interactions, etc. Commonly, a single noncovalent interaction is not as strong as covalent bonding. Nevertheless, the combination of noncovalent interactions as well as covalent bonding is an effective way develop tissue adhesives with a strong adhesive strength.

### 2.2. Strategies for Improving Cohesive Strength

**Figure 6:**
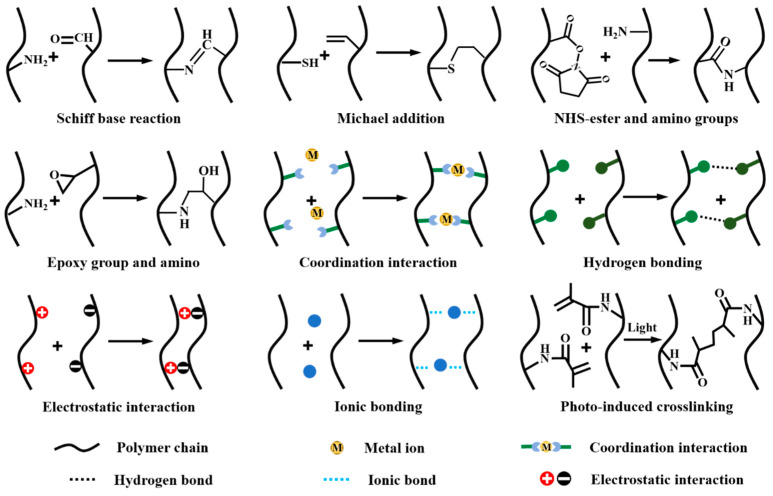
Solidification strategies of tissue adhesives. The strategies include classic covalent reactions, coordination crosslinking, physical crosslinking, and photo-induced crosslinking.

Cohesive strength is another factor influencing the adhesive performance of tissue adhesives. Over the past few decades, a variety of strategies have been proposed to enhance the cohesive strength of tissue adhesives, mainly including in situ covalent reactions, coordination crosslinking, physical crosslinking, and photo-induced crosslinking (Figure 6) [51,52]. In situ covalent crosslinking is the most commonly used crosslinking method. This crosslinking strategy utilizes classic organic reactions between the functional groups in raw or modified biodegradable polymer chains to crosslink the biodegradable polymers. The organic reactions mainly include a Schiff-base reaction (between amino and aldehyde groups) [53], a Michael addition (between sulfydryl group and double bond) [54], a reaction between NHS-ester/carboxyl and amino groups [1,55], or epoxy groups and aminos [56], etc. Since some functional groups, such as amino and sulfydryl groups, already exist or can be introduced in biodegradable polymers, in situ covalent crosslinking can be easily carried out. 

Coordination crosslinking typically occurs between functional groups (such as carboxyl and catechol) and metal ions [57]. Due to the dynamic nature of most coordinate bonds, coordination-crosslinked tissue adhesives usually show self-healing properties [57]. In addition, according to the type of metal ions, the resultant tissue adhesive can exhibit other desirable functions, such as antibacterial properties [58], conductivity [59], and the ability to promote wound healing [60]. Noncovalent bond-mediated physical crosslinking is also widely used in the solidification of tissue adhesives. The most used physical crosslinking among biodegradable polymers is based on metal coordination, H-bonding, ionic bonding, and electrostatic interactions, etc. [48]. Noncovalent bond-mediated physical crosslinking often occurs in mild conditions, and, therefore, this type of gelation is suitable for loading cells or protein drugs [61]. 

By introducing polymerizable double bonds (methacrylamide or methacrylate) [62], the biodegradable polymer can also be photo-crosslinked. This type of crosslinking can be controlled by light intensity and the irradiation time [63]. In addition, some tissue adhesives are crosslinked through a combination of multiple crosslinking strategies, such as the combination of ionic and covalent crosslinking [63], Schiff base and coordination crosslinking [15], and Schiff-base crosslinking and ionic crosslinking [64]. A multiply crosslinked tissue adhesive can show the advantages of each crosslinking type, and due to an increase in crosslinking density, the resultant tissue adhesive usually exhibits a better cohesive strength than singly crosslinked adhesives [15,64]. 

## 3. Biodegradable Polymers for Tissue Adhesives

Both natural and synthetic biodegradable polymers can be used to develop tissue adhesives. The typical advantages and drawbacks of natural or synthetic polymers are summarized in Table 2. In the following, we will provide an overview of natural or synthetic biodegradable polymer-based tissue adhesives, with the specific focus on the degradability and applications of these tissue adhesives.

### 3.1. Natural Polymers

#### 3.1.1. Chitosan

**Figure 7:**
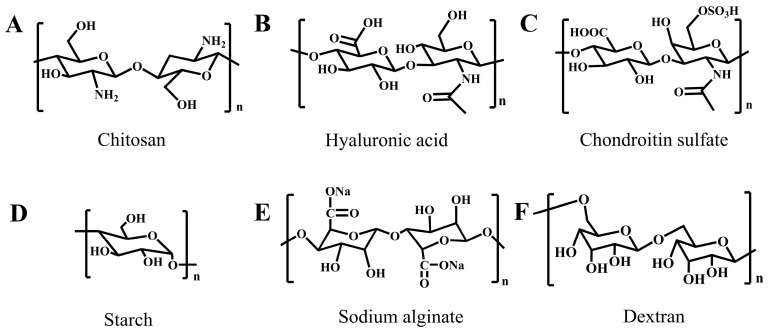
Chemical structures of some natural biodegradable polymers for tissue adhesives. (**A**) Chitosan; (**B**) hyaluronic acid; (**C**) chondroitin; (**D**) starch; (**E**) sodium alginate; (**F**) dextran.

Chitosan is a biodegradable, naturally occurring polymer with low toxicity and immunogenicity [65]. Currently, chitosan is mainly produced by the deacetylation of chitin under alkaline conditions [66,67]. The structural units of chitosan contain a majority of -NH_2_ groups (Figure 7A). The abundance of -NH_2_ groups allows for chitosan to be easily modified through reactions between amino and carboxyl groups or aldehyde [68,69]. Therefore, chitosan and its derivatives are widely used biodegradable polymers in developing biodegradable tissue adhesives. 

The repeated units of chitosan are connected to each other by a β-1,4-glycosidic bond. The degradation of chitosan is generated by the hydrolysis of this bond in the presence of enzymes such as lysozyme, amylase, pepsin, and other chitosan enzymes in organisms. After the hydrolysis, chitosan is converted to chito-oligosaccharides, which can enter biological cells with the assistance of glucose transporter proteins [70]. With the help of glucoamylase enzymes, the chito-oligosaccharides are further degraded and finally converted to H_2_O and CO_2_ through the metabolic systems of the organisms [71].

Due to the biodegradability of chitosan, usually, chitosan-based tissue adhesives are also biodegradable. For example, Rao and coworkers developed a carboxymethyl chitosan-polydopamine (FCMCS-PDA)-based hydrogel adhesive through a Schiff-base reaction and hydrogen bonding, and they found that the FCMCS-PDA adhesive degraded slowly in vitro. After 21 days of incubation in a phosphate buffer solution (PBS, pH = 7.4), a weight loss of about ~8.4%–~12.8% was observed [72]. Adding an enzyme to the PBS can significantly accelerate the degradation of chitosan-based tissue adhesives. Jin and coworkers developed a chitosan-based adhesive through enzymatic crosslinking and found that applying lysozyme to the adhesive (1 wt%) resulted in about 60% degradation after 21 days [73]. In addition, chitosan-based tissue adhesives are also found to be degradable in vivo. Singh and coworkers prepared a quaternized and phosphorylated chitosan/tannic acid-based hydrogel adhesive through physical crosslinking. After subcutaneously implanting the adhesive into the dorsal side of rats, the researchers found that only 64.0% of the adhesive was residual on day 7, and the adhesive was fully degraded after 14 days [74].

By virtue of their biocompatibility and biodegradability, chitosan-based tissue adhesives have been widely investigated and applied in wound closures and healing. For example, Zhao and coworkers prepared an injectable chitosan-g-polyaniline (QCSP)/benzaldehyde group functionalized poly(ethylene glycol)-co-poly(glycerol sebacate) (PEGS-FA) hydrogel adhesive through a Schiff-base reaction. They found that the adhesive hydrogel exhibited multiple attractive properties, mainly including tissue adhesiveness, a free radical-scavenging capacity, a self-healing ability, antibacterial activity, and biocompatibility and that it can significantly promote wound healing in vivo [75]. In addition, Chen and coworkers developed a chitosan/oxidized konjac glucomannan hydrogel adhesive by a Schiff-base reaction. The hydrogel adhesive exhibited tissue adhesiveness, self-healing properties, as well as biocompatibility and could promote wound healing after its application in a full-thickness skin defect [76].

Another important application of chitosan-based tissue adhesives is hemorrhage control. Due to its inherent cationic properties, chitosan can cause the aggregation of negatively charged erythrocyte and platelets, which makes it suitable for hemostasis. Liu and coworkers developed an injectable thermosensitive chitosan/glycerophosphate hydrogel. It was also found that injection of the gel at the bleeding site of rat livers completely stopped the bleeding within 2 min [77].

In addition, chitosan possesses an antibacterial ability, enabling them to work in the treatment of infected wounds. At lower concentrations, protonated amino groups on chitosan can interact with negatively charged microbial membranes, thereby altering the permeability and morphology of their membranes and leading to the death of bacteria [66]. Cui and coworkers developed a quaternized chitosan (QCS)/dialdehyde terminated polyethylene glycol (PEGDA) tissue adhesive by a Schiff-base reaction. The QCS/PEGDA tissue adhesive exhibited a strong antibacterial ability and could promote infected-wound healing. On day 9, the hydrogel-treated wound was almost healed, and the area of the wound treated by erythromycin ointment (ERY) still remained about 20% [78].

A diabetic wound is usually difficult to heal. One important reason is that high blood sugar leads to the excessive accumulation of reactive oxygen species (ROS) and a loss of the inherent protective function of skin, producing the exudation of tissue fluids from wounds. This provides conditions for the growth of pathogens and leads to wound infections [79]. Chitosan-based tissue adhesives with antimicrobial activity, therefore, can facilitate diabetic-wound healing. Peng and coworkers developed a chitosan grafted-dihydrocaffeic acid (CS-DA)/benzaldehyde-terminated Pluronic F127 (PF127-CHO)-based adhesive through a Schiff-base reaction. The adhesive exhibited antibacterial abilities (killing ratios for *S. aureus* (>80%) and *E. coil* (>40%)) and can promote the healing of diabetic wounds. After 14 days, the diabetic wounds treated by the hydrogel were close to healing, while the wounds in the control group (the Tegaderm membrane group) remained unclosed [80].

#### 3.1.2. Hyaluronic Acid

Hyaluronic acid, a natural, non-sulfated glycosaminoglycan, is the main component of the skin extracellular matrix (ECM) [81,82]. Linear hyaluronic acid is composed of D-glucuronic acid and *N*-acetylglucosamine-based repeated units, which are linked by a β-1,3-glycosidic bond and a β-1,4-glycosidic bond (Figure 7B) [83]. There are many hydroxy and carboxyl groups in hyaluronic acid, endowing it with high hydrophilicity [84]. Additionally, the abundant groups also make it easy to introduce functional groups in hyaluronic acid, which improves its tissue adhesiveness. For example, to enhance tissue adhesion, aldehyde groups and catechol groups are often required to be introduced to hyaluronic acid. Due to the existence of a hydroxy group, the aldehyde group can be easily obtained by NaIO_4_-mediated oxidation [85], and catechol groups can be introduced by a coupling reaction between the amino of dopamine and carboxyl in hyaluronic acid [86]. 

As a component of the ECM, hyaluronic acid is also biodegradable, and its degradation in vivo is mainly through a hyaluronidase (HYALs)-mediated degradation by breaking the β-1,4 glycosidic bond [84,87]. HYALs include HYAL-1 and HYAL-2. Typically, HYAL-2 breaks hyaluronic acid into fragments with a molecular weight of 20 kDa. The fragments with a relatively small molecular weight are internalized by cells and are further degraded by HYAL-1 in the lysosome [88]. In addition, hyaluronic acid also undergoes reactive oxygen species (ROS)-mediated oxidized degradation [89]. During the occurrence of some tissue wounds and inflammation, large amounts of ROS produced in the body degrades hyaluronic acid. ROS-mediated degradation occurs also by breaking the β-1,3 glycosidic bond [90].

Due to the biodegradability of hyaluronic acid, hyaluronic acid-based tissue adhesives also show good biodegradability. Hao and coworkers synthesized a poly(butynyl phospholane)-random-poly(ethylethylene phosphate)/hyaluronic acid adhesive through a thiol-yne “click” reaction and found that this tissue adhesive can be gradually degraded after HYAL treatment. After 34 days, the weight loss reached 91.2% ± 6.6% [91]. The concentration of HYALs is also found to influence the degradation of hyaluronic acid-based tissue adhesives. For example, Gwon and coworkers prepared a Si-based nickel oxide nanoflower (Si@NiO)-incorporated, visible-light-crosslinked hyaluronic acid tissue adhesive (Si@NiO/HA) and demonstrated that the degradation rate of this Si@NiO/HA adhesive was measured to be 1.2%, 1.4%, 1.5%, and 2.2% when placed in solutions containing 10, 50, 100, and 500 IU/mL of HYALs, respectively. Additionally, the sensitivity of tissue adhesives to HYALs is another factor influencing degradation. The control adhesive without Si@NiO (HA adhesive) was completely degraded within 12 days, whereas the degradation ratio of the Si@NiO/HA adhesive was less than 25%. The slow degradation was mainly because the incorporation of Si@NiO into the adhesive affected the sensitivity of the Si@NiO/HA adhesive to HYALs [92].

Due to the existence of HYALs in vivo, hyaluronic acid-based tissue adhesives are also degradable in vivo even without the addition of HYALs. Zhang and coworkers developed a cyclic *o*-nitrobenzyl-modified hyaluronic acid (HA-CNB) tissue adhesive by photo-crosslinking. They found that the HA-CNB adhesive completely vanished within 2 days after implanting it into oral mucosal defects of pigs [93]. The in vivo degradation of hyaluronic acid-based tissue adhesives was also verified by Weiss’s group. They developed an in situ-formed hyaluronic acid (Si-HA) hydrogel adhesive and found that the low silanized HA adhesive completely degraded within 21 days after it was injected into the backs of mice [94].

Due to their biodegradability and biocompatibility, hyaluronic acid-based tissue adhesives have been widely used in various biomedical fields. The most basic application of hyaluronic acid-based tissue adhesives is wound closure and hemostasis [8,95]. These applications require hyaluronic acid-based tissue adhesives to exhibit a strong adhesive performance. To improve the adhesive performance of hyaluronic acid-based adhesives, many functional groups which benefit adhesion are introduced. For example, Wei and coworkers introduced an aldehyde group into hyaluronic acid by oxidation and developed a quaternized carboxymethyl chitosan (QCMCS)/oxidized hyaluronic acid (HA-CHO)-based adhesive by Schiff-base crosslinking. The introduction of the aldehyde group improved the tissue adhesion properties of the adhesive, benefiting the treatment of skin wounds [96].

Another important application of hyaluronic acid-based tissue adhesives is drug or cell delivery. Drug delivery is a common application of almost all hydrogels [97]. By the controlled release of specific drugs through a hyaluronic acid hydrogel, the target disease can be better treated. Compared with drug delivery, cell delivery is more difficult because the carrier material may be incompatible with cells [98]. It is difficult to promote cell proliferation, migration, and differentiation [99]. However, cell delivery is quite important and can be applied to tissue engineering, such as cartilage regeneration, corneal repair, and tendon healing [100]. Due to the presence of cell surface receptors, such as, RHAMM, ICAM-1, and CD44 [101], hyaluronic acid is suitable for cell delivery. For example, Shin and coworkers prepared hyaluronic acid-catechol (HA-CA) hydrogels by oxidative crosslinking. They found that the HA-CA hydrogels can be used as a good carrier of human adipose-derived stem cells (hADSCs) and human hepatocytes (hHEPs), showing promise in minimal cell therapy [102]. 

As hyaluronic acid is the main component of the ECM and can be used for stem cell culture, hyaluronic acid-based tissue adhesives can also promote tissue regeneration [103]. Chen and coworkers prepared an aldehyde- and methacrylate-modified hyaluronic acid tissue adhesive (AHAMA). In vitro cell culture experiments showed that the AHAMA hydrogel could promote the proliferation of bone marrow stem cells. The implantation of the AHAMA hydrogel in a rat cartilage defect model could effectively promote cartilage tissue regeneration [104].

By the incorporation of other functional nanomaterials, hyaluronic acid-based tissue adhesives can exhibit many desirable properties and, therefore, are suitable for some specific applications. For example, the incorporation of zinc oxide (ZnO) nanoparticles into a hyaluronic acid-based hydrogel adhesive can promote the antibacterial ability of the adhesive [105], and the incorporation of Ti_3_C_2_ MXene nanosheets benefits the proliferation of human umbilical vein endothelial cells, endowing the adhesive with the ability of promoting diabetic-wound healing [106].

#### 3.1.3. Gelatin

Gelatin is the product of the partial hydrolysis of collagen in connective tissues such as animal skin, bones, and muscle membranes [107]. Due to the different hydrolysis processes of collagen, the physicochemical properties of gelatin are different, which makes gelatin exhibit different structures and relative molecular weights [108]. Gelatin contains abundant amino and carboxyl groups. These groups are reactive and can be used to introduce adhesive groups such as phenol [109] and catechol [110,111] to enhance tissue adhesion or crosslinking-related groups such as methacrylamide [112,113] and methacrylate [114] to facilitate the curing of gelatin-based adhesives. As a proteinaceous natural polymer, gelatin is biodegradable [115], and its degradation in vivo is mainly caused by matrix metalloproteinases (MMPs) produced by fibroblasts, macrophages, neutrophils, etc. [116,117].

Gelatin-based tissue adhesives are also found to be biodegradable. Xu and coworkers prepared a genipin-crosslinked gelatin/chitosan hydrogel adhesive. They found that the gelatin/chitosan hydrogel adhesive can be degraded in a PBS solution and that the addition of collagenase significantly accelerated this degradation [118]. Since gelation-based tissue adhesives are usually multicomponent, their degradation is also influenced by other incorporated polymers. For example, Yang and coworkers synthesized a tissue adhesive based on catechol-modified gelatin, Fe^3+^, and a synthetic polymer that was susceptible to degradation under alkaline conditions [110]. Influenced by the synthetic polymer, they found that the multicomponent adhesive degraded rapidly under alkaline conditions (pH = 9) rather than a physiological environment (pH = 7.4). In in vivo environments, gelatin-based tissue adhesives can also be degraded due to the existence of MMPs in vivo. For example, Wang and coworkers synthesized an injectable gelatin-based tissue adhesive (QCN@Gel-Zr) by coordination crosslinking. After 7 days of a subcutaneous injection of the adhesive, most of the adhesive was found to be degraded [119].

Gelatin-based adhesives have been applied in various biomedical fields, such as hemorrhage control [120], tumor therapy [121], wound treatments [122], and a controlled drug release [123]. Additionally, gelatin-based adhesives were also found to be able to stimulate cell growth, thus promoting tissue regeneration [124]. For example, Sharifi and coworkers designed a gelatin-glycidyl methacrylate adhesive (GELGYM) for corneal tissue regeneration. After 48 h of incubation in the GELGYM adhesive, corneal fibroblasts (HCFs), corneal endothelial cells (HCEns), human corneal epithelial cells (HCEps), and mixed neuroblastoma cells (NDCs) all maintained nearly 100% activity and underwent spreading, migration, and proliferation. In addition, the GELGYM adhesive can close corneal defects and withstand pressures of up to 200 mmHg, which allows for the adhesive to better adhere to corneal defects and promote corneal tissue regeneration [114].

#### 3.1.4. Chondroitin Sulfate

Chondroitin sulfate, with the repeat unit of β-1,3-linked *N*-acetyl galactosamine and β-1,4-linked glucuronic acid, is an important component of the cartilaginous ECM (Figure 7C) [125,126]. As a natural polymer, chondroitin sulfate can be enzymatically degraded in vivo. In the presence of chondroitinase ABC (ChABC), the chondroitin is first degraded to oligosaccharides by breaking the β-1,4-glycosidic bond between disaccharide repeating units [127]. The resultant oligosaccharides then enter cells in the presence of glucose transporter proteins and are finally catabolized by glucoamylase [128]. In addition, chondroitin can also be degraded by HYALs, which catalytically degrade chondroitin sulfate [129].

Due to the biodegradability of chondroitin sulfate, chondroitin sulfate-based tissue adhesives are also found to be degradable. Shin and coworkers developed a catechol-modified chondroitin (CS-CA) tissue adhesive and showed that the hydrogel can be degraded in vitro in the presence of chondroitinase that was produced from human intestinal anaerobic bacteria. In addition, they found that the degradation rate was influenced by the concentrations of catechol groups. The CS-CA tissue adhesive, with excessive catechol groups, showed a dense gel network, which limited the cleavage sites on the main chain by chondroitinase and finally resulted in a slower degradation rate [126]. The degradation of chondroitin-based tissue adhesives was also verified by Bryant’s group, who developed a mesenchymal stem cell (MSC)-loaded tissue adhesive with matrix metalloproteinase 7 (MMP7)-sensitive compounds as crosslinkers. As MSCs differentiate into chondrocytes, the matrix metalloproteinase was expressed. The in vitro degradation results show that the enzyme degraded the crosslinker, leading to the sustained degradation of the hydrogel adhesive [130].

Since chondroitin sulfate is the component of the cartilaginous ECM, apart from wound treatments [131], chondroitin sulfate-based tissue adhesives are also widely applied in promoting cartilage regeneration [132]. For example, Han and coworkers prepared a polydopamine-chondroitin sulfate-polyacrylamide (PDA-CS-PAM) tissue adhesive and implanted it into the defect of a cartilage. The polydopamine and chondroitin sulfate components in the hydrogel synergistically regulate cell behaviors and induce cartilage regeneration, offering a potential growth-factor-free solution for cartilage repair [133].

#### 3.1.5. Starch 

Starch is one of the most abundant biodegradable natural polymers and a polysaccharide found in plants [134]. It is composed of many repeating monosaccharides linked by α-1,4-glycosidic or α-1,6-glycosidic bonds (Figure 7D). Starch is classified into amylose and amylopectin. Amylose is an unbranched helical structure, which results in low water solubility. Amylopectin has a branched structure, whose main chain consists of glucose residues linked by α-1,4-glycosidic bonds, and the branched chain is linked by α-1,6-glycosidic bonds [135]. It has been reported that heating starch in water is a process in which starch granules absorb water, swell, break down their structure, and form a gel [136]. Compared to other biodegradable polymers, starch is abundant and cost-effective. Thus, starch-based hydrogel adhesives are widely used in various biological applications [137].

Starch is degradable in the body mainly by the amylase enzyme [138]. The amylase enzyme is widely distributed in the saliva and pancreas. The amylase enzyme in human body fluids is mainly the α-amylase secreted by pancreas and salivary glands, which is the most important enzyme for hydrolyzing carbohydrates in the human body [139]. α-amylase is an endonuclease, which could degrade both amylose and amylopectin [140]. It unconditionally cleaves the α-1,4-glycosidic bond and is ineffective on the α-1.6-glycosidic bond. When amylose starch is degraded, the end product is mainly glucose, and when amylopectin is degraded, the end product includes α-dextrin. The glucose produced by degradation provides essential energy for the body [141].

Starch-based hydrogel adhesives can be degraded in an environment containing α-amylase [17]. In an environment without enzymes, the hydrolysis of crosslinked bonds can also lead to the degradation of starch-based hydrogel adhesives. For example, Dong and coworkers synthesized a starch-based hydrogel by a Michael-type “thiol-ene” addition reaction between dithiol-functionalized poly(ethylene glycol) (PEG-SH) and acyl-modified sulfobetaine-derived starch (SB-ST-A). Due to the hydrolysis of ester that formed in the crosslinking process, they found that the weight of the hydrogel decreased dramatically after 9 days in PBS (pH = 7.4), and after 21 days, the hydrogel was completely degraded [142]. In addition, starch-based hydrogels could also be degraded in vivo. Ye and coworkers developed a starch-based physical hydrogel by the self-assembly of a starch-graft-poly(sulfobetaine methacrylate) (ST-g-PSBMA) through electrostatic interactions. After one week of subcutaneous implantation, the dimensions of the hydrogels gradually decreased, and after 4 weeks of implantation, the hydrogels were apparently degraded to fragments [143].

With good biocompatibility and biodegradability, starch-based tissue adhesives have been widely studied and applied in hemostasis. For example, Mao and coworkers developed a starch-based adhesive hydrogel (GPAH) by ionic crosslinking. They evaluated the hemostatic and wound-sealing performance of the gel using a rat femoral artery injury model. It was found that the bleeding mass (2.4 ± 1.3 g), after sealing with the GPAH hydrogel, was significantly lower than that of the group sealed by gauze (6.3 ± 1.5 g), and no serious inflammatory reaction occurred [144]. Another application of starch-based hydrogel adhesives is cell delivery. Zhuang and coworkers developed a 3D-printed nanocomposite starch-based tissue adhesive that provides attachment points for cells to promote cell growth. They utilized the hydrogel adhesive to culture mouse embryo fibroblast (NIH 3T3) cells and found that the cultured cells showed excellent attachment and migration properties and a faster proliferation rate [145].

#### 3.1.6. Sodium Alginate

Sodium alginate is a natural polymer derived from brown algae. Its polymer chain consists of β-*D*-mannuronic acid and α-*L*-guluronic acid, which are linked by the 1,4-glycosidic bond (Figure 7E) [146]. Sodium alginate contains large amounts of -COO- and thus exhibits polyanionic behavior in an aqueous solution [147]. Therefore, sodium alginate can gel with most divalent cations. The most commonly studied divalent cation is Ca^2+^, because Ca^2+^ is non-toxic and is able to induce gelation rapidly in mild environments [148]. 

Sodium alginate-based hydrogel adhesives are also degradable. Their degradation can be controlled by the degree of oxidation of sodium alginate. For example, Gao and coworkers prepared oxidized sodium alginate hydrogels by Ca^2+^-mediated crosslinking. They found that the degradation rates of the hydrogels were different when their degree of oxidation varied. The in vitro degradation results show that the weight loss of the hydrogel with a 30% degree of oxidation was 80.27%, while the weight loss of the hydrogel with a 10% degree of oxidation was 73.00% in 180 min [149].

Similar to other polysaccharide-based tissue adhesives, sodium alginate-based tissue adhesives are also widely applied in wound closures and healing [150,151] and tissue repair [152,153] and have obtained desirable results. As an anionic polysaccharide, sodium alginate-based tissue adhesives can be used to achieve controlled drug release. In an acidic medium, the -COO^−^ of sodium alginate is converted to -COOH, which decreases the degree of ionization. The hydrophilicity of sodium alginate decreases, and the molecular chain contracts. As the pH increases, the carboxyl group dissociates continuously. Therefore, the hydrophilicity of sodium alginate increases, and the molecular chain stretches [154]. Based on this principle, sodium alginate-based tissue adhesives achieve the controlled release of drugs in different pHs. For example, Wu and coworkers developed a carboxymethyl chitosan/sodium alginate hydrogel adhesive for the controlled release of lidocaine (LID) drugs. Due to the different swelling ratios of hydrogel adhesives in different pHs, it was found that the released drug was more at pH = 7.4 than at pH = 1.2 [155].

#### 3.1.7. Dextran

Dextran is a polysaccharide that consists of dextrose as a monosaccharide, with a repeated dextrose unit linked by glycosidic bonds (Figure 7F) [156]. Dextran has been widely found in nature. The structure of dextran varies, as the conditions for generating dextran are different [157]. Dextran is biodegradable and can be degraded to dextrose with a small molecular weight by the β-glucanase enzyme that cleaves the glycosidic bonds between the dextrose units [158]. These small molecules of dextrose are decomposed into CO_2_ and H_2_O through the body’s metabolic system, which provides energy for the body. 

Dextran-based tissue adhesives are degradable both in vitro and in vivo [159,160]. This degradation was found to be dependent on the polymer content and the composition of dextran-based tissue adhesives. For example, Yang and coworkers developed a poly(lysine-cysteine) (EPL-SH)/oxidized dextran (ODex) hydrogel adhesive by a Schiff-base reaction. The in vitro degradation results show that the degradation rate increases with the decrease in the polymeric content. The hydrogels with 10 wt% and 15 wt% polymeric contents were completely degraded after 14 h and 20 h, respectively, while the degradation of the hydrogel with an 8 wt% polymer content was completed in 9 h [161]. In addition, Zhao and coworkers developed a photo-curable gelatin methacrylamide/oxidized dextran hydrogel adhesive. An in vitro degradation experiment showed that the time required for the complete degradation of this hydrogel increased as the mass content of gelatin methacrylamide and oxidized dextran increased. The difference in degradation was mainly due to different crosslinking densities [162].

Commonly, dextran-based tissue adhesives are widely applied in drug deliveries [163,164], tissue repair [165,166], and bioprinting [167,168]. By combining other functional polymers, dextran-based adhesives can obtain new functions. For example, Zhu and coworkers combined choline-phosphoryl-functionalized chitosan and oxidized dextran and developed a chitosan/dextran hydrogel adhesive by a Schiff-base reaction. They found that the hydrogel adhesive can promote the aggregation of erythrocyte, showing its potential application in wound hemostasis [169]. In addition, Wu and coworkers combined DP7 peptide, ceftazidime, and oxidized dextran and obtained a ceftazidime-loaded DP7-Odex hydrogel adhesive by a Schiff-base reaction. Due to the synergistic effect of the DP7 peptide and ceftazidime in antibiotic properties, the ceftazidime-loaded DP7-Odex adhesive exhibited antimicrobial properties and can promote wound repair [170].

#### 3.1.8. Pectin

Pectin is a heteropolysaccharide widely found in plant cell walls, which mainly consists of α-1, 4 *D*-galacturonic acid units [171,172]. Pectin is relatively stable in acidic solutions than in alkaline solutions and can be divided into high ester pectin and low ester pectin based on the degree of pectin esterification. High ester pectin forms a non-reversible gel in the range of a soluble sugar content of ≥60% and pH = 2.6–3.4. Low ester pectin is unaffected by sugar and acid, because a part of methyl ester is transformed into primary amide. However, it requires divalent ions such as Ca^2+^ and Mg^2+^ to form a gel [173]. 

Pectin is a biodegradable, non-toxic natural polymer that can be used as a tissue adhesive. Although pectinase, which is required for pectin degradation, does not exist in the human body, some reports have shown that some pectin-based tissue adhesives are degradable in vivo [174]. Nowadays, the mechanism of degradation or clearance of pectin in vivo is still unclear. To facilitate a better degradation of pectin-based hydrogel adhesives, degradable crosslinkers can be introduced to crosslink the tissue adhesive. Pereira and coworkers utilized a biscysteine matrix-metalloproteinase (MMP)-cleavable peptide as the crosslinker and formed a pectin-based hydrogel by a photo-click reaction [175]. They found that the prepared pectin hydrogel was degraded in the presence of collagenase (type II). Since collagenase is present in vivo, the results indicate that the MMP-cleavable peptide-crosslinked pectin-based hydrogel has potential in degradation in vivo. 

Similar to other biodegradable polymer-based tissue adhesives, pectin-based adhesives can be applied in hemostasis [176] and tissue repair [177]. In addition, pectin-based adhesives also show applications in cell and drug loading. Mehrali and coworkers developed a pectin methacrylate/thiolated gelatin hydrogel adhesive (PEMA) through UV-induced crosslinking and a Michael addition reaction [178]. They demonstrated that human mesenehymal stem cells (hMSCs) cultured in the hydrogel showed about 50% confluency and about a 90% survival rate after 14 days of incubation. Additionally, Bostanci and coworkers developed a methacrylated gelatin/methacrylated pectin hydrogel adhesive by UV crosslinking and used this adhesive to load antibacterial curcumin [179]. Due to the high solubility of curcumin and the higher swelling ratio of the hydrogel in a basic condition, the hydrogel adhesive released curcumin faster in the media at pH = 7.4 than at pH = 5.0. As the pH of skin with chronic wounds is between 6–8, the adhesive hydrogel showed promise in the treatment of chronic wounds.

#### 3.1.9. Fibrin Sealants

Fibrin glue is the most common fibrinogen-based protein tissue adhesive, which has been commercialized and used in clinics. Fibrin glue is composed of fibrinogen, thrombin, factor VIII, and calcium ions. It mimics the clotting process by activating fibrinogen through thrombin. Thrombin cleaves the fibrinogen polypeptide to form fibrin monomers, which then form fibrin polymers through hydrogen bonding. Factor VIII activated by thrombin further catalyzes the formation of crosslinks among the fibrin polymers [2]. Calcium ions are involved in all processes. Since most of the components of fibrin glue are found in living organisms, fibrin glue is biodegradable [2]. However, fibrin glue has the risk of transmitting blood disease, and its adhesive strength to biological tissue is low [2]. In addition to simulating blood coagulation processes, fibrinogen can also be modified to fabricate novel fibrinogen-based tissue adhesives. For example, Simaan-Yameen and coworkers modified fibrinogen with methacrylic anhydride and developed a methacrylated fibrinogen (FibMA) hydrogel adhesive by light-activated free-radical polymerization. The FibMA hydrogel was found to be degraded both in vitro and in vivo. Due to the biological attributes of fibrinogen, the FibMA hydrogel can be used as a scaffold for cells, showing great potential in tissue healing and regeneration [180]. 

In addition, some other protein-incorporated tissue adhesives have also been developed in recent years. These proteins contain phase-transferring lysozyme (PTL), TGF-β1-affinity peptide. Due to the desirable functions of these proteins, such as the function of the generation of phase transitions and the ability of TGF-β1 enrichment, these tissue adhesives are applied in multi-functional dressings [181] and cartilage tissue regeneration [182].

### 3.2. Synthetic Polymers 

#### 3.2.1. Poly(lactic acid)

**Figure 8:**
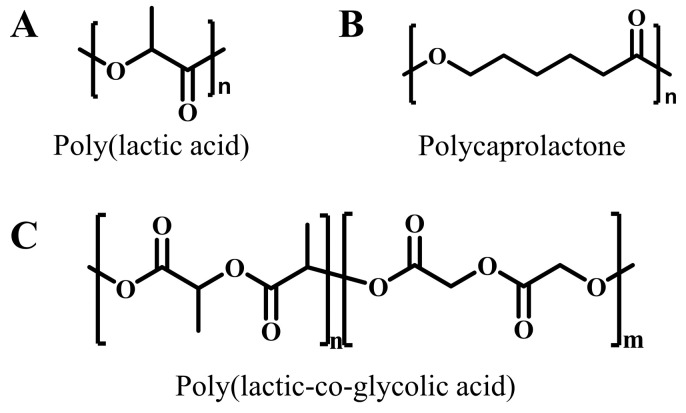
Chemical structure of some biodegradable polymers for tissue adhesives. (**A**) poly(lactic acid); (**B**) polycaprolactone; (**C**) poly(lactic-co-glycolic acid).

Poly(lactic acid) is a renewable, aliphatic synthetic polyester polymer (Figure 8A), which is produced from lactic acid through condensation polymerization (direct or azeotropic dehydration) or ring-opening polymerization [183]. Since lactic acid has *D*-type and *L*-type isomers, poly(lactic acid) is divided into three types: poly-*L*-lactic acid (PLLA), poly-*D*-lactic acid (PDLA), and poly-*D*, *L*-lactic acid (PDLLA) [184]. Poly(lactic acid) is biodegradable, and its degradation is mainly through hydrolysis, enzymatic degradation, and photodegradation. The hydrolytic degradation of poly(lactic acid) proceeds via random cleavage of the ester bond, which releases oligomers and monomers [185]. Enzymatic degradation occurs in the presence of poly(lactic acid)-degrading enzymes, such as proteases and lipases. For example, the activated carbonyl group in proteinase K can react with the ester group of poly(lactic acid), resulting in the cleavage of the ester bond [186]. Photodegradation is initiated by photoionization, which can break the poly(lactic acid) chains. 

The degradation of poly(lactic acid) is affected by pH due to the different cleavage modes under different pHs. Under acidic conditions, the mode of degradation is a chain-end cleavage, whereas under alkaline conditions, the degradation takes place by random ester cleavages [187]. Poly(lactic acid)-based hydrogel adhesives were found to be degraded faster under alkaline conditions. Jang and coworkers utilized fluorescence changes to confirm the fast degradation under alkaline conditions. They synthesized a hydrogel microcarrier based on the diacrylate copolymer of poly(lactic acid) and polyethylene glycol, which can be photo-crosslinked by UV irradiation. The results of an in vitro degradation show that the degradation of the hydrogel produced less fluorescence changes at pH = 5 than at pH = 7.4, indicating that the hydrogel degrades faster in pH = 7.4 than pH = 5 [188]. 

Based on the good biodegradability and histocompatibility of poly(lactic acid), poly(lactic acid)-based hydrogel adhesives have been applied in three-dimensional bioresorbable scaffolds [189], the prevention of post-operative abdominal adhesions [190], and biomimetic coatings [191]. In addition, recent results also show that poly(lactic acid)-based hydrogel adhesives can be used for a controlled drug release. For example, Grindy and coworkers developed a polyethylene glycol- poly(lactic acid) hydrogel system for a sustained drug release. They found that altering the ratio of the hydrophilic polyethylene glycol to a hydrophobic poly(lactic acid) in the hydrogel, or crosslinking the polyethylene glycol and poly(lactic acid) chains, could modulate the kinetics of drug release to maximize the therapeutic effect of the drug [192].

#### 3.2.2. Polyurethanes

Polyurethane (PU) is characterized by the presence of urethane bonds and can be easily synthesized by addition reactions between alcohols and isocyanates [193]. Due to its high chemical inertness, chemical stability, flexibility, and environmental stability, polyurethane has been applied in the fields of adhesives, biomedicine, and tissue engineering [194]. Polyurethane can be enzymatically degraded or hydrolyzed. The enzymes that have been identified to degrade polyurethane are of microbial origin but also come from mammalian cells, such as porcine pancreatic lipase, or from plants, such as papain from papaya [195]. 

The degradation of polyurethane-based hydrogels is mainly through the hydrolysis of the ester group of polyurethane. Reducing the hydrophilicity or increasing the crosslinking density of polyurethane-based hydrogels can lower the degradation rate. For example, Feng and coworkers synthesized a hydrophobic curcumin-composited polyurethane hydrogel (PU-CUR). An in vitro degradation experiment showed that the incorporation of curcumin reduced the degradation rate due to the reduction in the hydrophilicity and the increase in the crosslinking density of the hydrogels [196]. In addition, the hydrogel containing polyurethane was also shown to be degradable in vivo. Wang and coworkers mixed a fluorescent polyurethane emulsion containing tetraphenyl ethylene with oxidized dextran to form a polyurethane-based hydrogel by a dynamic acylhydrazone bond. They showed that the remaining weight of the hydrogel was only about 20% of the initial weight after 96 h of subcutaneous implantation [197]. Similar to other biodegradable polymers, PU-based adhesives are mainly applied in hemostasis [198], wound healing [199], cell and drug delivery [200,201,202], and tissue regeneration [203].

### 3.3. Others

Polycaprolactone is a polymer prepared via the ring opening polymerization of ε-caprolactone monomers (Figure 8B) [204]. In an aqueous medium or in vivo, polycaprolactone is degraded mainly by hydrolysis. When polycaprolactone comes into contact with water, it undergoes hydrolysis, and the ester group is broken, which results in the decomposition of the polymer [205]. Polycaprolactone has excellent biocompatibility and biodegradability. Therefore, polycaprolactone-based tissue adhesives are also biodegradable and can be used in many biomedical fields, such as cell delivery [206], drug delivery [207], and tissue regeneration [208]. 

Poly(lactic-co-glycolic acid) is a biodegradable polymer formed by the random polymerization of a lactic acid monomer and hydroxyacetic acid (Figure 8C) [209,210,211]. Breaking the cyclic ester group can lead to the degradation of poly(lactic-co-glycolic acid), and the degradation products are lactic acid and hydroxyacetic acid, which are also by-products of human metabolism [210]. The degradation rate of poly(lactic-co-glycolic acid) can be adjusted by controlling the ratio of lactic acid and hydroxyacetic acid. On the one hand, if the lactic acid content exceeds 30%, the synthesized poly(lactic-co-glycolic acid) tends to be amorphous and is degraded slowly. On the other hand, when the glycolic acid proportion increases, the hydrophilicity of the polymer increases, which accelerates the degradation. In addition, pH also influences the degradation. Poly(lactic-co-glycolic acid) degrades faster under alkaline conditions, because oligomeric units of poly(lactic-co-glycolic acid) have higher solubility under alkaline conditions [212]. With their biocompatibility and degradability, poly(lactic-co-glycolic acid)-incorporated hydrogel adhesives are applied in wound dressings [213], cartilage defect repair [214], and burn-wound healing [215].

## 4. Challenges and Perspectives of Biodegradable Polymer-Based Tissue Adhesives

Biodegradable polymers, either natural or synthetic ones, are degradable under specific conditions. However, their degradation performances in vivo are usually different. Some natural polymers, such as hyaluronic acid, gelatin, starch, and chondroitin sulfate, degrade well under in vivo conditions due to the presence of related enzymes in vivo [84,116,129,138]. Synthetic biodegradable polymers such as poly(lactic acid) and polycaprolactone, which degrade through hydrolysis, can also be slowly degraded in the body [185,205]. However, for sodium alginate and pectin, there is an absence of enzymes that can degrade them in vivo. Therefore, the in vivo degradation of these polymers is difficult, and when these polymers are used to design tissue adhesives, other biodegradable polymers are usually required [174]. 

Rationally choosing biodegradable polymers for specific applications is essential. Due to the different inherent characteristics, some polymers are more promising in specific areas of application. For example, due to its cationic properties, chitosan can cause the aggregation of erythrocyte and platelets, making it more suitable for hemostasis [216]. Hyaluronic acid is a main component of the ECM, and, therefore, hyaluronic acid is more promising for cell delivery than other polymers [82,124]. As a component of the cartilaginous ECM, chondroitin sulfate is promising in promoting cartilage regeneration [132].

Although tissue adhesives exhibit attractive properties and are promising in numerous biomedical fields, there are still some challenges for current biodegradable polymer-based tissue adhesives. First, the design of tissue adhesives with a degradation rate matching the rate of wound healing is quite challenging. Commonly, the slow degradation of tissue adhesives causes the delayed healing of wounds, and fast degradation makes adhesives lose their structure and functions in an untimely fashion. Therefore, tissue adhesives with a suitable degradation rate which matches the wound healing rate are highly desired. However, the degradation rate of biodegradable polymer-based tissue adhesives is hard to precisely control. The molecular weight and hydrophilicity of biodegradable polymers [217], the crosslinking density of tissue adhesives [2], and the microenvironment of injury tissues [11] are all found to influence the degradation. Therefore, future studies should focus on comprehensively considering these factors, rather than merely using biodegradable polymers to form degradable tissue adhesives.

Secondly, a detailed characterization of the degradation of biodegradable polymer-based tissue adhesives in vivo is difficult. Currently, the characterization of the degradation of tissue adhesives is mainly dependent on the direct observation of size changes through naked eyes [118,119] or through the detection of the fluorescent intensity of fluorescence-labeled tissue adhesives [218]. These methods can only roughly estimate the degradation of tissue adhesives. Some valuable molecule-level changes of biodegradable polymers and tissue adhesives in vivo are difficult to directly detect. More efforts can be made in developing advanced detection technologies for degradation in vivo.

In addition, commonly, in vitro degradation experiments can provide a reference for the degradation fate of the implanted material in vivo. However, in vivo environments, this is more complicated. Due to different degradation conditions in vitro and in vivo, in vitro degradation experiments can hardly accurately predict the degradation properties of the material in vivo. For example, it has been reported that an in situ-forming hyaluronic acid hydrogel fully degraded in 3 days in vitro, but it required 21 days to degrade the same tissue adhesive in vivo [94]. The large difference between the degradation behaviors in vitro and in vivo is also a challenge for investigating the degradation behavior of tissue adhesives.

## 5. Conclusions

Tissue adhesives have emerged as a promising alternative tool in wound treatments due to their advantages in ease of use, rapid application, less pain, and minimal tissue damage. To endow tissue adhesives with degradability, a variety of biodegradable polymers have been utilized to develop tissue adhesives. These biodegradable polymers can be divided into natural polymers, such as chitosan, hyaluronic acid, gelatin, chondroitin sulfate, starch, sodium alginate, glucans, pectin, and functional proteins, as well as synthetic polymers, such as poly (lactic acid), polyurethanes, polycaprolactone, and poly(lactic-co-glycolic acid). In this review, we summarized the strategies for developing tissue adhesives by utilizing biodegradable polymers. Further, we provided an overview of every type of biodegradable polymer-based tissue adhesive, with a focus on their degradation behaviors and specific applications. The challenges and perspectives of biodegradable polymer-constructed tissue adhesives were also discussed. We expect that a deeper understanding biodegradable polymer-based tissue adhesives could further accelerate the clinical transformation of the various kinds of tissue adhesives.

## Figures and Tables

**Table 1:** Representative biodegradable polymer-based commercial tissue adhesives [2,7].

Main Component	Natural or Synthetic	Trade Name
Chitosan	Natural	Polysaccharide HemCon Bandage Pro
Alginate	Natural	Seal-V
Hyaluronic acid	Natural	Seprafilm Adhesion Barrier
Gelatin	Natural	LifeSeal GRF Biological Glue
Collagen	Natural	Angio-Seal
Human fibrinogen	Natural	Tisseel Evicel Hemaseel Crosseal VISTASEAL CryoSeal Fibrin Sealant System
Polyurethane	Synthetic	MAR-CUTIS (Flix)

**Table 2:** Typical advantages and drawbacks of natural and synthetic polymers.

Polymers	Advantages	Drawbacks
Natural polymers	High biocompatibility in most cases; Suitable biodegradability; Non-toxic degradation products.	Relatively weak mechanical properties; Restricted sources; High production costs.
Synthetic polymers	Low cost; Easy to produce massively; Adjustable mechanical properties.	Lack of biological cues; Relatively poor biodegradability in most cases.

## Data Availability

Not applicable.

## References

[1] Bouten P.J.M., Zonjee M., Bender J., Yauw S.T.K., van Goor H., van Hest J.C.M., Hoogenboom R. (2014). The chemistry of tissue adhesive materials. Prog. Polym. Sci..

[2] Nam S., Mooney D. (2021). Polymeric tissue adhesives. Chem. Rev..

[3] Smeets R., Tauer N., Vollkommer T., Gosau M., Henningsen A., Hartjen P., Fruh L., Beikler T., Sturmer E.K., Rutkowski R. (2022). Tissue adhesives in reconstructive and aesthetic surgery-application of silk fibroin-based biomaterials. Int. J. Mol. Sci..

[4] Bal-Ozturk A., Cecen B., Avci-Adali M., Topkaya S.N., Alarcin E., Yasayan G., Ethan Y.C., Bulkurcuoglu B., Akpek A., Avci H. (2021). Tissue adhesives: From research to clinical translation. Nano Today.

[5] Chen G., Zhao Y. (2020). Omnipotent tissue adhesive. Sci. Bull..

[6] Ren H., Zhang Z., Chen X., He C. (2024). Stimuli-responsive hydrogel adhesives for wound closure and tissue regeneration. Macromol. Biosci..

[7] Xu K., Wu X., Zhang X., Xing M. (2022). Bridging wounds: Tissue adhesives’ essential mechanisms, synthesis and characterization, bioinspired adhesives and future perspectives. Burn. Trauma.

[8] Han G.Y., Kwack H.W., Kim Y.H., Je Y.H., Kim H.J., Cho C.S. (2024). Progress of polysaccharide-based tissue adhesives. Carbohydr. Polym..

[9] Modaresifar K., Azizian S., Hadjizadeh A. (2016). Nano/biomimetic tissue adhesives development: From research to clinical application. Polym. Rev..

[10] Ghobril C., Grinstaff M.W. (2015). The chemistry and engineering of polymeric hydrogel adhesives for wound closure: A tutorial. Chem. Soc. Rev..

[11] Taboada G.M., Yang K.S., Pereira M.J.N., Liu S.H.S., Hu Y.S., Karp J.M., Artzi N., Lee Y.H. (2020). Overcoming the translational barriers of tissue adhesives. Nat. Rev. Mater..

[12] Bao Z., Gao M., Sun Y., Nian R., Xian M. (2020). The recent progress of tissue adhesives in design strategies, adhesive mechanism and applications. Mater. Sci. Eng. C Mater. Biol. Appl..

[13] Han G.Y., Hwang S.K., Cho K.H., Kim H.J., Cho C.S. (2023). Progress of tissue adhesives based on proteins and synthetic polymers. Biomater. Res..

[14] Duarte A.P., Coelho J.F., Bordado J.C., Cidade M.T., Gil M.H. (2012). Surgical adhesives: Systematic review of the main types and development forecast. Prog. Polym. Sci..

[15] Li S.D., Chen N., Li X.P., Li Y., Xie Z.P., Ma Z.Y., Zhao J., Hou X., Yuan X.B. (2020). Bioinspired double-dynamic-bond crosslinked bioadhesive enables post-wound closure care. Adv. Funct. Mater..

[16] He X.Y., Sun A., Li T., Qian Y.J., Qian H., Ling Y.F., Zhang L.H., Liu Q.Y., Peng T., Qian Z. (2020). Mussel-inspired antimicrobial gelatin/chitosan tissue adhesive rapidly activated in situ by H_2_O_2_/ascorbic acid for infected wound closure. Carbohydr. Polym..

[17] Cui R., Chen F., Zhao Y., Huang W., Liu C. (2020). A novel injectable starch-based tissue adhesive for hemostasis. J. Mater. Chem. B.

[18] Zhao X., Huang Y., Li Z., Chen J., Luo J., Bai L., Huang H., Cao E., Yin Z., Han Y. (2024). Injectable self-expanding/self-propelling hydrogel adhesive with procoagulant activity and rapid gelation for lethal massive hemorrhage management. Adv. Mater..

[19] Koivusalo L., Kauppila M., Samanta S., Parihar V.S., Ilmarinen T., Miettinen S., Oommen O.P., Skottman H. (2019). Tissue adhesive hyaluronic acid hydrogels for sutureless stem cell delivery and regeneration of corneal epithelium and stroma. Biomaterials.

[20] Day N.B., Dalhuisen R., Loomis N.E., Adzema S.G., Prakash J., Shields Iv C.W. (2022). Tissue-adhesive hydrogel for multimodal drug release to immune cells in skin. Acta Biomater..

[21] Xue W., Shi W., Kuss M., Kong Y., Alimi O.A., Wang H., DiMaio D.J., Yu C., Duan B. (2023). A dual-network nerve adhesive with enhanced adhesion strength promotes transected peripheral nerve repair. Adv. Funct. Mater..

[22] Cui C.Y., Wu T.L., Chen X.Y., Liu Y., Li Y., Xu Z.Y., Fan C.C., Liu W.G. (2020). A janus hydrogel wet adhesive for internal tissue repair and anti-postoperative adhesion. Adv. Funct. Mater..

[23] Wu D., Shi X., Zhao F., Chilengue S.T.F., Deng L., Dong A., Kong D., Wang W., Zhang J. (2019). An injectable and tumor-specific responsive hydrogel with tissue-adhesive and nanomedicine-releasing abilities for precise locoregional chemotherapy. Acta Biomater..

[24] Kim M.K., Moon Y.A., Song C.K., Baskaran R., Bae S., Yang S.G. (2018). Tumor-suppressing miR-141 gene complex-loaded tissue-adhesive glue for the locoregional treatment of hepatocellular carcinoma. Theranostics.

[25] Zhao Y., Song S.L., Ren X.Z., Zhang J.M., Lin Q., Zhao Y.L. (2022). Supramolecular adhesive hydrogels for tissue engineering applications. Chem. Rev..

[26] Mahdavi A., Ferreira L., Sundback C., Karp J.M. (2008). A biodegradable and biocompatible gecko-inspired tissue adhesive. Proc. Natl. Acad. Sci. USA.

[27] Ge L.P., Chen S.X. (2020). Recent advances in tissue adhesives for clinical medicine. Polymers.

[28] Bhagat V., Becker M.L. (2017). Degradable adhesives for surgery and tissue engineering. Biomacromolecules.

[29] Griffith L.G. (2002). Emerging design principles in biomaterials and scaffolds for tissue engineering. Ann. N. Y. Acad. Sci..

[30] Zhang L.W., Zhang Y.J., Ma F.S., Liu X.Z., Liu Y.Z., Cao Y., Pei R.J. (2022). A low-swelling and toughened adhesive hydrogel with anti-microbial and hemostatic capacities for wound healing. J. Mater. Chem. B.

[31] Bian S.Q., Hao L.Z., Qiu X., Wu J., Chang H., Kuang G.M., Zhang S., Hu X.H., Dai Y.K., Zhou Z.Y. (2022). An injectable rapid-adhesion and anti-swelling adhesive hydrogel for hemostasis and wound sealing. Adv. Funct. Mater..

[32] Iliev G., Hardan L., Kassis C., Bourgi R., Cuevas-Suarez C.E., Lukomska-Szymanska M., Mancino D., Haikel Y., Kharouf N. (2021). Shelf life and storage conditions of universal adhesives: A literature review. Polymers.

[33] Song K.C., Hao Y.M., Liu Y., Cao R.F., Zhang X.L., He S.W., Wen J., Zheng W.S., Wang L.L., Zhang Y.J. (2023). Preparation of pectin-chitosan hydrogels based on bioadhesive-design micelle to prompt bacterial infection wound healing. Carbohydr. Polym..

[34] Liu S.D., Li D.S., Wang Y., Zhou G.Q., Ge K., Jiang L. (2023). Adhesive, antibacterial and double crosslinked carboxylated polyvinyl alcohol/chitosan hydrogel to enhance dynamic skin wound healing. Int. J. Biol. Macromol..

[35] Konieczynska M.D., Villa-Camacho J.C., Ghobril C., Perez-Viloria M., Tevis K.M., Blessing W.A., Nazarian A., Rodriguez E.K., Grinstaff M.W. (2016). On-demand dissolution of a dendritic hydrogel-based dressing for second-degree burn wounds through thiol-thioester exchange reaction. Angew. Chem. Int. Ed..

[36] Yang J., Yu H.J., Wang L., Liu J., Liu X.W., Hong Y.C., Huang Y.D., Ren S.N. (2022). Advances in adhesive hydrogels for tissue engineering. Eur. Polym. J..

[37] Wei C.Z., Song J.L., Tan H.Q. (2021). A paintable ophthalmic adhesive with customizable properties based on symmetrical/asymmetrical cross-linking. Biomater. Sci..

[38] Daristotle J.L., Erdi M., Lau L.W., Zaki S.T., Srinivasan P., Balabhadrapatruni M., Ayyub O.B., Sandler A.D., Kofinas P. (2021). Biodegradable, tissue adhesive polyester blends for safe, complete wound healing. ACS Biomater. Sci. Eng..

[39] Fan C.J., Fu J.Y., Zhu W.Z., Wang D.A. (2016). A mussel-inspired double-crosslinked tissue adhesive intended for internal medical use. Acta Biomater..

[40] Zeng Z.W., Liu D.H., Li D.J., Mo X.M. (2021). An injectable double cross-linked hydrogel adhesive inspired by synergistic effects of mussel foot proteins for biomedical application. Colloids Surf. B Biointerfaces.

[41] Fan P., Dong Q., Yang J., Chen Y., Yang H., Gu S., Xu W., Zhou Y. (2023). Flexible dual-functionalized hyaluronic acid hydrogel adhesives formed in situ for rapid hemostasis. Carbohydr. Polym..

[42] Liang J., Zhang K., Li J., Su J., Guan F., Li J. (2022). Injectable protocatechuic acid based composite hydrogel with hemostatic and antioxidant properties for skin regeneration. Mater. Des..

[43] Yang S.Y., O’Cearbhaill E.D., Sisk G.C., Park K.M., Cho W.K., Villiger M., Bouma B.E., Pomahac B., Karp J.M. (2013). A bio-inspired swellable microneedle adhesive for mechanical interlocking with tissue. Nat. Commun..

[44] Scognamiglio F., Travan A., Rustighi I., Tarchi P., Palmisano S., Marsich E., Borgogna M., Donati I., de Manzini N., Paoletti S. (2016). Adhesive and sealant interfaces for general surgery applications. J. Biomed. Mater. Res. B Appl. Biomater..

[45] Wu S.J., Zhao X. (2023). Bioadhesive technology platforms. Chem. Rev..

[46] Pethrick R.A. (2015). Design and ageing of adhesives for structural adhesive bonding—A review. Proc. Imech. E. Part L J. Mater. Des. Appl..

[47] Merotto E., Pavan P.G., Piccoli M. (2023). Three-dimensional bioprinting of naturally derived hydrogels for the production of biomimetic living tissues: Benefits and challenges. Biomedicines.

[48] Hoque M., Alam M., Wang S.R., Zaman J.U., Rahman M.S., Johir M.A.H., Tian L.M., Choi J.G., Ahmed M.B., Yoon M.H. (2023). Interaction chemistry of functional groups for natural biopolymer-based hydrogel design. Mater. Sci. Eng. R Rep..

[49] Li M., Pan G.Y., Zhang H.L., Guo B.L. (2022). Hydrogel adhesives for generalized wound treatment: Design and applications. J. Polym. Sci..

[50] Zhou D., Li S., Pei M., Yang H., Gu S., Tao Y., Ye D., Zhou Y., Xu W., Xiao P. (2020). Dopamine-modified hyaluronic acid hydrogel adhesives with fast-forming and high tissue adhesion. ACS Appl. Mater. Interfaces.

[51] Lin X., Zhao X.W., Xu C.Z., Wang L.L., Xia Y.Z. (2022). Progress in the mechanical enhancement of hydrogels: Fabrication strategies and underlying mechanisms. J. Polym. Sci..

[52] Hou X., Lin L., Li K., Jiang F., Qiao D., Zhang B., Xie F. (2024). Towards superior biopolymer gels by enabling interpenetrating network structures: A review on types, applications, and gelation strategies. Adv. Colloid Interface Sci..

[53] Li Z., Lin Z. (2021). Recent advances in polysaccharide-based hydrogels for synthesis and applications. Aggregate.

[54] Wang J., Niu J., Sawada T., Shao Z., Serizawa T. (2017). A bottom-up synthesis of vinyl-cellulose nanosheets and their nanocomposite hydrogels with enhanced strength. Biomacromolecules.

[55] Li J., Celiz A.D., Yang J., Yang Q., Wamala I., Whyte W., Seo B.R., Vasilyev N.V., Vlassak J.J., Suo Z. (2017). Tough adhesives for diverse wet surfaces. Science.

[56] Cengiz N. (2020). Glutathione-responsive multifunctionalizable hydrogels via amine-epoxy “click” chemistry. Eur. Polym. J..

[57] Yan G.H., Chen G.F., Peng Z.Q., Shen Z.L., Tang X., Sun Y., Zeng X.H., Lin L. (2021). The cross-linking mechanism and applications of catechol-metal polymer materials. Adv. Mate. Interfaces.

[58] Wahid F., Zhong C., Wang H.S., Hu X.H., Chu L.Q. (2017). Recent advances in antimicrobial hydrogels containing metal ions and metals/metal oxide nanoparticles. Polymers.

[59] Xu J., Jin R., Duan L., Ren X., Gao G. (2019). Tough, adhesive and conductive polysaccharide hydrogels mediated by ferric solution. Carbohydr. Polym..

[60] Ahmadian Z., Gheybi H., Adeli M. (2022). Efficient wound healing by antibacterial property: Advances and trends of hydrogels, hydrogel-metal np composites and photothermal therapy platforms. J. Drug Deliv. Sci. Technol..

[61] Liu H., Liu J., Qi C., Fang L., Zhuo R., Jiang X. (2016). Thermosensitive injectable in-situ forming carboxymethyl chitin hydrogel for three-dimensional cell culture. Acta Biomater..

[62] Samadian H., Maleki H., Allahyari Z., Jaymand M. (2020). Natural polymers-based light-induced hydrogels: Promising biomaterials for biomedical applications. Coord. Chem. Rev..

[63] Fonseca R.G., De Bon F., Pereira P., Carvalho F.M., Freitas M., Tavakoli M., Serra A.C., Fonseca A.C., Coelho J.F.J. (2022). Photo-degradable, tough and highly stretchable hydrogels. Mater. Today Bio..

[64] Vitkova L., Musilova L., Achbergerova E., Kolarik R., Mrlik M., Korpasova K., Mahelova L., Capakova Z., Mracek A. (2022). Formulation of magneto-responsive hydrogels from dually cross-linked polysaccharides: Synthesis, tuning and evaluation of rheological properties. Int. J. Mol. Sci..

[65] Hamedi H., Moradi S., Hudson S.M., Tonelli A.E., King M.W. (2022). Chitosan based bioadhesives for biomedical applications: A review. Carbohydr. Polym..

[66] Ardean C., Davidescu C.M., Nemes N.S., Negrea A., Ciopec M., Duteanu N., Negrea P., Duda-Seiman D., Musta V. (2021). Factors influencing the antibacterial activity of chitosan and chitosan modified by functionalization. Int. J. Mol. Sci..

[67] Pal K., Bharti D., Sarkar P., Anis A., Kim D., Chalas R., Maksymiuk P., Stachurski P., Jarzebski M. (2021). Selected applications of chitosan composites. Int. J. Mol. Sci..

[68] Wang W., Xue C., Mao X. (2020). Chitosan: Structural modification, biological activity and application. Int. J. Biol. Macromol..

[69] Sharma S., Jain P., Tiwari S. (2020). Dynamic imine bond based chitosan smart hydrogel with magnified mechanical strength for controlled drug delivery. Int. J. Biol. Macromol..

[70] Bhuvanachandra B., Sivaramakrishna D., Alim S., Preethiba G., Rambabu S., Swamy M.J., Podile A.R. (2021). New class of chitosanase from bacillus amyloliquefaciens for the generation of chitooligosaccharides. J. Agric. Food Chem..

[71] El-Sayed W.N., Alkabli J., Aloqbi A., Elshaarawy R.F.M. (2021). Optimization enzymatic degradation of chitosan into amphiphilic chitooligosaccharides for application in mitigating liver steatosis and cholesterol regulation. Eur. Polym. J..

[72] Rao K.M., Narayanan K.B., Uthappa U.T., Park P.H., Choi I., Han S.S. (2022). Tissue adhesive, self-healing, biocompatible, hemostasis, and antibacterial properties of fungal-derived carboxymethyl chitosan-polydopamine hydrogels. Pharmaceutics.

[73] Jin R., Teixeira L.S.M., Dijkstra P.J., Karperien M., van Blitterswijk C.A., Zhong Z.Y., Feijen J. (2009). Injectable chitosan-based hydrogels for cartilage tissue engineering. Biomaterials.

[74] Singh G., Nayal A., Malhotra S., Koul V. (2020). Dual functionalized chitosan based composite hydrogel for haemostatic efficacy and adhesive property. Carbohydr. Polym..

[75] Zhao X., Wu H., Guo B., Dong R., Qiu Y., Ma P.X. (2017). Antibacterial anti-oxidant electroactive injectable hydrogel as self-healing wound dressing with hemostasis and adhesiveness for cutaneous wound healing. Biomaterials.

[76] Chen H., Cheng J., Ran L., Yu K., Lu B., Lan G., Dai F., Lu F. (2018). An injectable self-healing hydrogel with adhesive and antibacterial properties effectively promotes wound healing. Carbohydr. Polym..

[77] Liu C., Liu C., Liu Z., Shi Z., Liu S., Wang X., Wang X., Huang F. (2023). Injectable thermogelling bioadhesive chitosan-based hydrogels for efficient hemostasis. Int. J. Biol. Macromol..

[78] Cui H., Cui B., Chen H., Geng X., Geng X., Li Z., Cao S., Shen J., Li J. (2023). A chitosan-based self-healing hydrogel for accelerating infected wound healing. Biomater. Sci..

[79] Wang C.W., Liu Y.H., Yang X.X., Li W.T., Zhou X.H., Ren Y., Zhang C.R., Yang H., Kong W.Q., Wang J.W. (2022). In-situ forming hydrogel incorporated with reactive oxygen species responsive and antibacterial properties for diabetic infected chronic wound healing. Chem. Eng. J..

[80] Peng H., Li H.C., Zhang X., Tang J.Z., Liang Y.P., Qiao L.P., Zhu Y., Hou M.M., Wei S.M., Zhang Z.X. (2023). 3D-exosomes laden multifunctional hydrogel enhances diabetic wound healing via accelerated angiogenesis. Chem. Eng. J..

[81] Graca M.F.P., Miguel S.P., Cabral C.S.D., Correia I.J. (2020). Hyaluronic acid-based wound dressings: A review. Carbohydr. Polym..

[82] Zhai P., Peng X., Li B., Liu Y., Sun H., Li X. (2020). The application of hyaluronic acid in bone regeneration. Int. J. Biol. Macromol..

[83] Luo Y., Tan J., Zhou Y., Guo Y., Liao X., He L., Li D., Li X., Liu Y. (2023). From crosslinking strategies to biomedical applications of hyaluronic acid-based hydrogels: A review. Int. J. Biol. Macromol..

[84] Amorim S., Reis C.A., Reis R.L., Pires R.A. (2021). Extracellular matrix mimics using hyaluronan-based biomaterials. Trends Biotechnol..

[85] Yu Z., Li Q., He X., Wang X., Wen Y., Zeng L., Yu W., Hu P., Chen H. (2023). A multifunctional hydrogel based on nature polysaccharide fabricated by Schiff base reaction. Eur. Polym. J..

[86] Ryu J.H., Hong S., Lee H. (2015). Bio-inspired adhesive catechol-conjugated chitosan for biomedical applications: A mini review. Acta Biomater..

[87] Schmaus A., Rothley M., Schreiber C., Moller S., Rosswag S., Franz S., Garvalov B.K., Thiele W., Spataro S., Herskind C. (2022). Sulfated hyaluronic acid inhibits the hyaluronidase cemip and regulates the ha metabolism, proliferation and differentiation of fibroblasts. Matrix Biol..

[88] Kilic E., Kilic G., Karadas G., Akgul O., Aytekin M., Sonmez M.F., Ozgocmen S. (2015). Serum and tissue levels of hyaluronan in patients with systemic sclerosis. Ann. Rheum. Dis..

[89] Juranek I., Stern R., Soltes L. (2014). Hyaluronan peroxidation is required for normal synovial function: An hypothesis. Med. Hypotheses.

[90] Berdiaki A., Neagu M., Spyridaki I., Kuskov A., Perez S., Nikitovic D. (2023). Hyaluronan and reactive oxygen species signaling-novel cues from the matrix?. Antioxidants.

[91] Hao Y., He J., Ma X., Feng L., Zhu M., Zhai Y., Liu Y., Ni P., Cheng G. (2019). A fully degradable and photocrosslinked polysaccharide-polyphosphate hydrogel for tissue engineering. Carbohydr. Polym..

[92] Gwon K., Park J.D., Lee S., Choi W.I., Hwang Y., Mori M., Yu J.S., Lee D.N. (2022). Injectable hyaluronic acid hydrogel encapsulated with si-based nio nanoflower by visible light cross-linking: Its antibacterial applications. Int. J. Biol. Macromol..

[93] Zhang W., Bao B., Jiang F., Zhang Y., Zhou R., Lu Y., Lin S., Lin Q., Jiang X., Zhu L. (2021). Promoting oral mucosal wound healing with a hydrogel adhesive based on a phototriggered s-nitrosylation coupling reaction. Adv. Mater..

[94] Flegeau K., Toquet C., Rethore G., d’Arros C., Messager L., Halgand B., Dupont D., Autrusseau F., Lesoeur J., Veziers J. (2020). In situ forming, silanized hyaluronic acid hydrogels with fine control over mechanical properties and in vivo degradation for tissue engineering applications. Adv. Healthc. Mater..

[95] Liang X.L., Huang C., Liu H., Chen H., Shou J.B., Cheng H.W., Liu G. (2023). Natural hydrogel dressings in wound care: Design, advances, and perspectives. Chin. Chem. Lett..

[96] Wei Q., Chen K., Zhang X., Ma G., Zhang W., Hu Z. (2022). Facile preparation of polysaccharides-based adhesive hydrogel with antibacterial and antioxidant properties for promoting wound healing. Colloids Surf. B. Biointerfaces.

[97] Khan F., Atif M., Haseen M., Kamal S., Khan M.S., Shahid S., Nami S.A.A. (2022). Synthesis, classification and properties of hydrogels: Their applications in drug delivery and agriculture. J. Mater. Chem. B.

[98] Říhová B. (2000). Immunocompatibility and biocompatibility of cell delivery systems. Adv. Drug Del. Rev..

[99] Zhao C., Tan A., Pastorin G., Ho H.K. (2013). Nanomaterial scaffolds for stem cell proliferation and differentiation in tissue engineering. Biotechnol. Adv..

[100] Yang J., Yamato M., Nishida K., Ohki T., Kanzaki M., Sekine H., Shimizu T., Okano T. (2006). Cell delivery in regenerative medicine: The cell sheet engineering approach. J. Control. Release.

[101] Slevin M., Krupinski J., Gaffney J., Matou S., West D., Delisser H., Savani R.C., Kumar S. (2007). Hyaluronan-mediated angiogenesis in vascular disease: Uncovering rhamm and cd44 receptor signaling pathways. Matrix Biol..

[102] Shin J., Lee J.S., Lee C., Park H.J., Yang K., Jin Y., Ryu J.H., Hong K.S., Moon S.H., Chung H.M. (2015). Tissue adhesive catechol-modified hyaluronic acid hydrogel for effective, minimally invasive cell therapy. Adv. Funct. Mater..

[103] Yazdani M., Shahdadfar A., Jackson C.J., Utheim T.P. (2019). Hyaluronan-based hydrogel scaffolds for limbal stem cell transplantation: A review. Cells.

[104] Chen J., Yang J., Wang L., Zhang X., Heng B.C., Wang D.A., Ge Z. (2021). Modified hyaluronic acid hydrogels with chemical groups that facilitate adhesion to host tissues enhance cartilage regeneration. Bioact. Mater..

[105] Shirzaei Sani E., Portillo-Lara R., Spencer A., Yu W., Geilich B.M., Noshadi I., Webster T.J., Annabi N. (2018). Engineering adhesive and antimicrobial hyaluronic acid/elastin-like polypeptide hybrid hydrogels for tissue engineering applications. ACS Biomater. Sci. Eng..

[106] Li Y., Fu R., Duan Z., Zhu C., Fan D. (2022). Artificial nonenzymatic antioxidant mxene nanosheet-anchored injectable hydrogel as a mild photothermal-controlled oxygen release platform for diabetic wound healing. ACS Nano.

[107] Mohanto S., Narayana S., Merai K.P., Kumar J.A., Bhunia A., Hani U., Al Fatease A., Gowda B.H.J., Nag S., Ahmed M.G. (2023). Advancements in gelatin-based hydrogel systems for biomedical applications: A state-of-the-art review. Int. J. Biol. Macromol..

[108] Alipal J., Mohd Pu’ad N.A.S.P., Lee T.C., Nayan N.H.M., Sahari N., Basri H., Idris M.I., Abdullah H.Z. (2021). A review of gelatin: Properties, sources, process, applications, and commercialisation. Mater. Today Proc..

[109] Liu Y., Cheong Ng S., Yu J., Tsai W.B. (2019). Modification and crosslinking of gelatin-based biomaterials as tissue adhesives. Colloids Surf. B. Biointerfaces.

[110] Yang Y., Shi K., Yu K., Xing F., Lai H., Zhou Y., Xiao P. (2022). Degradable hydrogel adhesives with enhanced tissue adhesion, superior self-healing, cytocompatibility, and antibacterial property. Adv. Healthc. Mater..

[111] Montazerian H., Mitra S., Najafabadi A.H., Seyedmahmoud R., Zheng Y.T., Dokmeci M.R., Annabi N., Khademhosseini A., Weiss P.S. (2023). Catechol conjugation for bioadhesion in photo-cross-linkable biomaterials. ACS Mater. Lett..

[112] Zhao X., Lang Q., Yildirimer L., Lin Z.Y., Cui W., Annabi N., Ng K.W., Dokmeci M.R., Ghaemmaghami A.M., Khademhosseini A. (2016). Photocrosslinkable gelatin hydrogel for epidermal tissue engineering. Adv. Healthc. Mater..

[113] Fang X., Xie J., Zhong L., Li J., Rong D., Li X., Ouyang J. (2016). Biomimetic gelatin methacrylamide hydrogel scaffolds for bone tissue engineering. J. Mater. Chem. B.

[114] Sharifi S., Islam M.M., Sharifi H., Islam R., Koza D., Reyes-Ortega F., Alba-Molina D., Nilsson P.H., Dohlman C.H., Mollnes T.E. (2021). Tuning gelatin-based hydrogel towards bioadhesive ocular tissue engineering applications. Bioact. Mater..

[115] Su K., Wang C. (2015). Recent advances in the use of gelatin in biomedical research. Biotechnol. Lett..

[116] Vandooren J., Van den Steen P.E., Opdenakker G. (2013). Biochemistry and molecular biology of gelatinase b or matrix metalloproteinase-9 (MMP-9): The next decade. Crit. Rev. Biochem. Mol. Biol..

[117] Loffek S., Schilling O., Franzke C.W. (2011). Series “matrix metalloproteinases in lung health and disease”: Biological role of matrix metalloproteinases: A critical balance. Eur. Respir. J..

[118] Xu Z.Y., Zhang H.H., Huang Y., Zhong H., Qin P.W., Cheng S.B., Wang Y.N., Yang C.H. (2023). Tough and biocompatible hydrogel tissue adhesives entirely based on naturally derived ingredients. ACS Appl. Polym. Mater..

[119] Wang C., Chen H., Wang W., Yan G., Zheng S., Wang C., Li N., Tang H. (2024). Facile strategy for gelatin-based hydrogel with multifunctionalities to remodel wound microenvironment and accelerate healing of acute and diabetic wounds. Int. J. Biol. Macromol..

[120] Hong Y., Zhou F., Hua Y., Zhang X., Ni C., Pan D., Zhang Y., Jiang D., Yang L., Lin Q. (2019). A strongly adhesive hemostatic hydrogel for the repair of arterial and heart bleeds. Nat. Commun..

[121] Jiang Y.A., Pan X.M., Yao M.Y., Han L., Zhang X., Jia Z.R., Weng J., Chen W.X., Fang L.M., Wang X.L. (2021). Bioinspired adhesive and tumor microenvironment responsive nanomofs assembled 3D-printed scaffold for anti-tumor therapy and bone regeneration. Nano Today.

[122] Liang Y., Zhao X., Hu T., Han Y., Guo B. (2019). Mussel-inspired, antibacterial, conductive, antioxidant, injectable composite hydrogel wound dressing to promote the regeneration of infected skin. J. Colloid Interface Sci..

[123] Gheysoori P., Paydayesh A., Jafari M., Peidayesh H. (2023). Thermoresponsive nanocomposite hydrogels based on Gelatin/poly (N-isopropylacrylamide) (PNIPAM) for controlled drug delivery. Eur. Polym. J..

[124] Park H., Temenoff J.S., Holland T.A., Tabata Y., Mikos A.G. (2005). Delivery of tgf-β1 and chondrocytes via injectable, biodegradable hydrogels for cartilage tissue engineering applications. Biomaterials.

[125] Zhao L., Liu M., Wang J., Zhai G. (2015). Chondroitin sulfate-based nanocarriers for drug/gene delivery. Carbohydr. Polym..

[126] Shin J., Kang E.H., Choi S., Jeon E.J., Cho J.H., Kang D., Lee H., Yun I.S., Cho S.W. (2021). Tissue-adhesive chondroitin sulfate hydrogel for cartilage reconstruction. ACS Biomater. Sci. Eng..

[127] Yang J., Shen M., Wen H., Luo Y., Huang R., Rong L., Xie J. (2020). Recent advance in delivery system and tissue engineering applications of chondroitin sulfate. Carbohydr. Polym..

[128] Hiraoka S., Furuichi T., Nishimura G., Shibata S., Yanagishita M., Rimoin D.L., Superti-Furga A., Nikkels P.G., Ogawa M., Katsuyama K. (2007). Nucleotide-sugar transporter slc35d1 is critical to chondroitin sulfate synthesis in cartilage and skeletal development in mouse and human. Nat. Med..

[129] Jedrzejas M.J., Stern R. (2005). Structures of vertebrate hyaluronidases and their unique enzymatic mechanism of hydrolysis. Annu. Rev. Food Sci. Technol..

[130] Aisenbrey E.A., Bryant S.J. (2018). A MMP7-sensitive photoclickable biomimetic hydrogel for msc encapsulation towards engineering human cartilage. J. Biomed. Mater. Res. A.

[131] Wu G., Ma F., Xue Y., Peng Y., Hu L., Kang X., Sun Q., Ouyang D.F., Tang B., Lin L. (2022). Chondroitin sulfate zinc with antibacterial properties and anti-inflammatory effects for skin wound healing. Carbohydr. Polym..

[132] Basu A., Kunduru K.R., Abtew E., Domb A.J. (2015). Polysaccharide-based conjugates for biomedical applications. Bioconjug. Chem..

[133] Han L., Wang M., Li P., Gan D., Yan L., Xu J., Wang K., Fang L., Chan C.W., Zhang H. (2018). Mussel-inspired tissue-adhesive hydrogel based on the polydopamine-chondroitin sulfate complex for growth-factor-free cartilage regeneration. ACS Appl. Mater. Interfaces.

[134] Yu X.P., Chen L., Jin Z.Y., Jiao A.Q. (2021). Research progress of starch-based biodegradable materials: A review. J. Mater. Sci..

[135] Junejo S.A., Flanagan B.M., Zhang B., Dhital S. (2022). Starch structure and nutritional functionality—Past revelations and future prospects. Carbohydr. Polym..

[136] BeMiller J.N. (2011). Pasting, paste, and gel properties of starch-hydrocolloid combinations. Carbohydr. Polym..

[137] Qamruzzaman M., Ahmed F., Mondal M.I.H. (2021). An overview on starch-based sustainable hydrogels: Potential applications and aspects. J. Polym. Environ..

[138] Dhital S., Warren F.J., Butterworth P.J., Ellis P.R., Gidley M.J. (2017). Mechanisms of starch digestion by alpha-amylase-structural basis for kinetic properties. Crit. Rev. Food Sci. Nutr..

[139] Peyrot des Gachons C., Breslin P.A. (2016). Salivary amylase: Digestion and metabolic syndrome. Curr. Diab. Rep..

[140] Liu P., Ma L., Duan W., Gao W., Fang Y., Guo L., Yuan C., Wu Z., Cui B. (2023). Maltogenic amylase: Its structure, molecular modification, and effects on starch and starch-based products. Carbohydr. Polym..

[141] Klip A., De Bock K., Bilan P.J., Richter E.A. (2024). Transcellular barriers to glucose delivery in the body. Annu. Rev. Physiol..

[142] Dong D., Li J., Cui M., Wang J., Zhou Y., Luo L., Wei Y., Ye L., Sun H., Yao F. (2016). In situ “clickable” zwitterionic starch-based hydrogel for 3D cell encapsulation. ACS Appl. Mater. Interfaces.

[143] Ye L., Zhang Y., Wang Q., Zhou X., Yang B., Ji F., Dong D., Gao L., Cui Y., Yao F. (2016). Physical cross-linking starch-based zwitterionic hydrogel exhibiting excellent biocompatibility, protein resistance, and biodegradability. ACS Appl. Mater. Interfaces.

[144] Mao Y.X., Li P., Yin J.W., Bai Y.J., Zhou H., Lin X., Yang H.L., Yang L. (2021). Starch-based adhesive hydrogel with gel-point viscoelastic behavior and its application in wound sealing and hemostasis. J. Mater. Sci. Technol..

[145] Zhuang P., Greenberg Z., He M. (2021). Biologically enhanced starch bio-ink for promoting 3D cell growth. Adv. Mater. Technol..

[146] Zia K.M., Zia F., Zuber M., Rehman S., Ahmad M.N. (2015). Alginate based polyurethanes: A review of recent advances and perspective. Int. J. Biol. Macromol..

[147] Ahmad A., Mubarak N.M., Jannat F.T., Ashfaq T., Santulli C., Rizwan M., Najda A., Bin-Jumah M., Abdel-Daim M.M., Hussain S. (2021). A critical review on the synthesis of natural sodium alginate based composite materials: An innovative biological polymer for biomedical delivery applications. Processes.

[148] Cai Y., Lu Q., Guo X., Wang S., Qiao J., Jiang L. (2015). Salt-tolerant superoleophobicity on alginate gel surfaces inspired by seaweed (saccharina japonica). Adv. Mater..

[149] Gao C.M., Liu M.Z., Chen J., Zhang X. (2009). Preparation and controlled degradation of oxidized sodium alginate hydrogel. Polym. Degrad. Stabil..

[150] Cao B., Wang C., Guo P., Zhang Q., Wang C., Sun H., Wen H., Chen X., Wang Y., Wang Y. (2023). Photo-crosslinked enhanced double-network hydrogels based on modified gelatin and oxidized sodium alginate for diabetic wound healing. Int. J. Biol. Macromol..

[151] Zhang J., Zhang S., Liu C., Lu Z., Li M., Hurren C., Wang D. (2024). Photopolymerized multifunctional sodium alginate-based hydrogel for antibacterial and coagulation dressings. Int. J. Biol. Macromol..

[152] Yuan H., Zheng X., Liu W., Zhang H., Shao J., Yao J., Mao C., Hui J., Fan D. (2020). A novel bovine serum albumin and sodium alginate hydrogel scaffold doped with hydroxyapatite nanowires for cartilage defects repair. Colloids Surf. B. Biointerfaces.

[153] Zhu Y., Kong L., Farhadi F., Xia W., Chang J., He Y., Li H. (2019). An injectable continuous stratified structurally and functionally biomimetic construct for enhancing osteochondral regeneration. Biomaterials.

[154] Yao B., Ni C., Xiong C., Zhu C., Huang B. (2010). Hydrophobic modification of sodium alginate and its application in drug controlled release. Bioprocess Biosyst. Eng..

[155] Wu M., Lin M., Li P., Huang X., Tian K., Li C. (2023). Local anesthetic effects of lidocaine-loaded carboxymethyl chitosan cross-linked with sodium alginate hydrogels for drug delivery system, cell adhesion, and pain management. J. Drug Deliv. Sci. Technol..

[156] Kagimura F.Y., da Cunha M.A., Barbosa A.M., Dekker R.F., Malfatti C.R. (2015). Biological activities of derivatized D-glucans: A review. Int. J. Biol. Macromol..

[157] Díaz-Montes E. (2021). Dextran: Sources, structures, and properties. Polysaccharides.

[158] Mejia S.M.V., de Francisco A., Bohrer B. (2020). A comprehensive review on cereal beta-glucan: Extraction, characterization, causes of degradation, and food application. Crit. Rev. Food Sci. Nutr..

[159] Zhao B., Zhang Y., Li D., Mo X., Pan J. (2022). Hofmeister effect-enhanced gelatin/oxidized dextran hydrogels with improved mechanical properties and biocompatibility for wound healing. Acta Biomater..

[160] Chen Z., Wu H., Wang H., Zaldivar-Silva D., Aguero L., Liu Y., Zhang Z., Yin Y., Qiu B., Zhao J. (2021). An injectable anti-microbial and adhesive hydrogel for the effective noncompressible visceral hemostasis and wound repair. Mater. Sci. Eng. C Mater. Biol. Appl..

[161] Yang R., Xue W., Liao H., Wu F., Guo H., Zhang W., Wang P., Tan X., Xu H., Chi B. (2022). Injectable polylysine and dextran hydrogels with robust antibacterial and ros-scavenging activity for wound healing. Int. J. Biol. Macromol..

[162] Zhao X., Li S., Du X., Li W., Wang Q., He D., Yuan J. (2022). Natural polymer-derived photocurable bioadhesive hydrogels for sutureless keratoplasty. Bioact. Mater..

[163] Zhang X., Nan K., Zhang Y., Song K., Geng Z., Shang D., Guan X., Fan L. (2024). A novel injectable hydrogel prepared from phenylboronic acid modified gelatin and oxidized-dextran for bone tissue engineering. Int. J. Biol. Macromol..

[164] Guan S., Zhang K., Cui L., Liang J., Li J., Guan F. (2022). Injectable gelatin/oxidized dextran hydrogel loaded with apocynin for skin tissue regeneration. Biomater. Adv..

[165] Pan J.F., Yuan L., Guo C.A., Geng X.H., Fei T., Fan W.S., Li S., Yuan H.F., Yan Z.Q., Mo X.M. (2014). Fabrication of modified dextran-gelatin in situ forming hydrogel and application in cartilage tissue engineering. J. Mater. Chem. B.

[166] Wang X., Li Z., Shi T., Zhao P., An K., Lin C., Liu H. (2017). Injectable dextran hydrogels fabricated by metal-free click chemistry for cartilage tissue engineering. Mater. Sci. Eng. C Mater. Biol. Appl..

[167] Li Z., Liu L., Chen Y. (2022). Direct 3D printing of thermosensitive aop127-oxidized dextran hydrogel with dual dynamic crosslinking and high toughness. Carbohydr. Polym..

[168] Musilova L., Achbergerova E., Vitkova L., Kolarik R., Martinkova M., Minarik A., Mracek A., Humpolicek P., Pecha J. (2022). Cross-linked gelatine by modified dextran as a potential bioink prepared by a simple and non-toxic process. Polymers.

[169] Zhu Z., Zhang K., Xian Y., He G., Pan Z., Wang H., Zhang C., Wu D. (2023). A choline phosphoryl-conjugated chitosan/oxidized dextran injectable self-healing hydrogel for improved hemostatic efficacy. Biomacromolecules.

[170] Wu S., Yang Y., Wang S., Dong C., Zhang X., Zhang R., Yang L. (2022). Dextran and peptide-based ph-sensitive hydrogel boosts healing process in multidrug-resistant bacteria-infected wounds. Carbohydr. Polym..

[171] Lara-Espinoza C., Carvajal-Millan E., Balandran-Quintana R., Lopez-Franco Y., Rascon-Chu A. (2018). Pectin and pectin-based composite materials: Beyond food texture. Molecules.

[172] Zdunek A., Pieczywek P.M., Cybulska J. (2021). The primary, secondary, and structures of higher levels of pectin polysaccharides. Compr. Rev. Food Sci. Food Saf..

[173] Cui Y.L., Chen J.F., Zhang S.G. (2022). The effect of degree of esterification of pectin on the interaction between pectin and wheat gluten protein. Food Hydrocoll..

[174] An H., Yang Y., Zhou Z., Bo Y., Wang Y., He Y., Wang D., Qin J. (2021). Pectin-based injectable and biodegradable self-healing hydrogels for enhanced synergistic anticancer therapy. Acta Biomater..

[175] Pereira R.F., Barrias C.C., Bartolo P.J., Granja P.L. (2018). Cell-instructive pectin hydrogels crosslinked via thiol-norbornene photo-click chemistry for skin tissue engineering. Acta Biomater..

[176] Wang J.H., Tsai C.W., Tsai N.Y., Chiang C.Y., Lin R.S., Pereira R.F., Li Y.E. (2021). An injectable, dual crosslinkable hybrid pectin methacrylate (pecma)/gelatin methacryloyl (gelma) hydrogel for skin hemostasis applications. Int. J. Biol. Macromol..

[177] Chen F., Ni Y., Liu B., Zhou T., Yu C., Su Y., Zhu X., Yu X., Zhou Y. (2017). Self-crosslinking and injectable hyaluronic acid/rgd-functionalized pectin hydrogel for cartilage tissue engineering. Carbohydr. Polym..

[178] Mehrali M., Thakur A., Kadumudi F.B., Pierchala M.K., Cordova J.A.V., Shahbazi M.A., Mehrali M., Pennisi C.P., Orive G., Gaharwar A.K. (2019). Pectin methacrylate (PEMA) and gelatin-based hydrogels for cell delivery: Converting waste materials into biomaterials. ACS Appl. Mater. Interfaces.

[179] Bostanci N.S., Buyuksungur S., Hasirci N., Tezcaner A. (2022). pH responsive release of curcumin from photocrosslinked pectin/gelatin hydrogel wound dressings. Biomater. Adv..

[180] Simaan-Yameen H., Bar-Am O., Saar G., Seliktar D. (2023). Methacrylated fibrinogen hydrogels for 3D cell culture and delivery. Acta Biomater..

[181] Chen J., Xu M., Wang L., Li T., Li Z., Wang T., Li P. (2022). Converting lysozyme to hydrogel: A multifunctional wound dressing that is more than antibacterial. Colloids Surf. B. Biointerfaces.

[182] Guo Q., Yin W., Wang H., Gao J., Gu Y., Wang W., Liu C., Pan G., Li B. (2024). Dynamic proteinaceous hydrogel enables in-situ recruitment of endogenous TGF-β1 and stem cells for cartilage regeneration. Adv. Funct. Mater..

[183] Hu Y., Daoud W.A., Cheuk K.K.L., Lin C.S.K. (2016). Newly developed techniques on polycondensation, ring-opening polymerization and polymer modification: Focus on poly(lactic acid). Materials.

[184] Li G., Zhao M., Xu F., Yang B., Li X., Meng X., Teng L., Sun F., Li Y. (2020). Synthesis and biological application of polylactic acid. Molecules.

[185] Elsawy M.A., Kim K.H., Park J.W., Deep A. (2017). Hydrolytic degradation of polylactic acid (PLA) and its composites. Renew. Sust. Energ. Rev..

[186] Qi X., Ren Y.W., Wang X.Z. (2017). New advances in the biodegradation of poly(lactic) acid. Int. Biodeterior. Biodegrad..

[187] Gesine Schliecker C.S. (2003). Stefan Fuchs, Thomas Kissel, Characterization of a homologous series of d,l-lactic acid oligomers; a mechanistic study on the degradation kinetics in vitro. Biomaterials.

[188] Jang W., Mun S.J., Kim S.Y., Bong K.W. (2023). Controlled growth factor delivery via a degradable poly(lactic acid) hydrogel microcarrier synthesized using degassed micromolding lithography. Colloids Surf. B. Biointerfaces.

[189] Pasini C., Pandini S., Re F., Ferroni M., Borsani E., Russo D., Sartore L. (2023). New poly(lactic acid)-hydrogel core-shell scaffolds highly support mscs’ viability, proliferation and osteogenic differentiation. Polymers.

[190] Fu S.Z., Li Z., Fan J.M., Meng X.H., Shi K., Qu Y., Yang L.L., Wu J.B., Fan J., Luot F. (2014). Biodegradable and thermosensitive monomethoxy poly(ethylene glycol)-poly(lactic acid) hydrogel as a barrier for prevention of post-operative abdominal adhesion. J. Biomed. Nanotechnol..

[191] Schneider M., Gunter C., Taubert A. (2018). Co-deposition of a hydrogel/calcium phosphate hybrid layer on 3d printed poly(lactic acid) scaffolds via dip coating: Towards automated biomaterials fabrication. Polymers.

[192] Grindy S., Gil D., Suhardi J., Fan Y., Moore K., Hugard S., Leape C., Randolph M., Asik M.D., Muratoglu O. (2023). Hydrogel device for analgesic drugs with in-situ loading and polymerization. J. Control. Release.

[193] Akindoyo J.O., Beg M.D.H., Ghazali S., Islam M.R., Jeyaratnam N., Yuvaraj A.R. (2016). Polyurethane types, synthesis and applications—A review. Rsc Adv..

[194] Wendels S., Averous L. (2021). Biobased polyurethanes for biomedical applications. Bioact. Mater..

[195] Magnin A., Pollet E., Phalip V., Averous L. (2020). Evaluation of biological degradation of polyurethanes. Biotechnol. Adv..

[196] Feng Y., Xiao K.C., He Y.Y., Du B.H., Hong J.H., Yin H., Lu D., Luo F., Li Z., Li J.H. (2021). Tough and biodegradable polyurethane-curcumin composited hydrogel with antioxidant, antibacterial and antitumor properties. Mater. Sci. Eng. C.

[197] Wang X., Ou Y., Wang X., Yuan L., He N., Li Z., Luo F., Li J., Tan H. (2023). A biodegradable injectable fluorescent polyurethane-oxidized dextran hydrogel for non-invasive monitoring. J. Mater. Chem. B.

[198] Zou F., Wang Y., Zheng Y., Xie Y., Zhang H., Chen J., Hussain M.I., Meng H., Peng J. (2022). A novel bioactive polyurethane with controlled degradation and l-arg release used as strong adhesive tissue patch for hemostasis and promoting wound healing. Bioact. Mater..

[199] Rai A., Ferrao R., Marta D., Vilaca A., Lino M., Rondao T., Ji J., Paiva A., Ferreira L. (2022). Antimicrobial peptide-tether dressing able to enhance wound healing by tissue contact. ACS Appl. Mater. Interfaces.

[200] Cheng Q.P., Hsu S.H. (2023). A self-healing hydrogel and injectable cryogel of gelatin methacryloyl-polyurethane double network for 3D printing. Acta Biomater..

[201] Polo Fonseca L., Trinca R.B., Felisberti M.I. (2018). Amphiphilic polyurethane hydrogels as smart carriers for acidic hydrophobic drugs. Int. J. Pharm..

[202] Laurano R., Boffito M., Abrami M., Grassi M., Zoso A., Chiono V., Ciardelli G. (2021). Dual stimuli-responsive polyurethane-based hydrogels as smart drug delivery carriers for the advanced treatment of chronic skin wounds. Bioact. Mater..

[203] Werkmeister J.A., Adhikari R., White J.F., Tebb T.A., Le T.P., Taing H.C., Mayadunne R., Gunatillake P.A., Danon S.J., Ramshaw J.A. (2010). Biodegradable and injectable cure-on-demand polyurethane scaffolds for regeneration of articular cartilage. Acta Biomater..

[204] Thielemans M.L.W. (2009). Synthesis of polycaprolactone: A review. Chem. Soc. Rev..

[205] Thakur M., Majid I., Hussain S., Nanda V. (2021). Poly(ε-caprolactone): A potential polymer for biodegradable food packaging applications. Packag. Technol. Sci..

[206] Salehi A.O.M., Keshel S.H., Sefat F., Tayebi L. (2021). Use of polycaprolactone in corneal tissue engineering: A review. Mater. Today Commun..

[207] Deng H., Dong A., Song J., Chen X. (2019). Injectable thermosensitive hydrogel systems based on functional peg/pcl block polymer for local drug delivery. J. Control Release.

[208] Patel M., Koh W.G. (2020). Composite hydrogel of methacrylated hyaluronic acid and fragmented polycaprolactone nanofiber for osteogenic differentiation of adipose-derived stem cells. Pharmaceutics.

[209] Lu Y., Cheng D., Niu B., Wang X., Wu X., Wang A. (2023). Properties of poly (lactic-co-glycolic acid) and progress of poly (lactic-co-glycolic acid)-based biodegradable materials in biomedical research. Pharmaceuticals.

[210] Kapoor D.N., Bhatia A., Kaur R., Sharma R., Kaur G., Dhawan S. (2015). PLGA: A unique polymer for drug delivery. Ther. Deliv..

[211] Kuperkar K., Atanase L.I., Bahadur A., Crivei I.C., Bahadur B. (2024). Degradable polymeric bio(nano)materials and their biomedical applications: A comprehensive overview and recent Updates. Polymers.

[212] Zolnik B S., Burgess D J. (2007). Effect of acidic pH on PLGA microsphere degradation and release. J. Control. Release.

[213] Ma W., Zhou M., Dong W., Zhao S., Wang Y., Yao J., Liu Z., Han H., Sun D., Zhang M. (2021). A bi-layered scaffold of a poly(lactic-co-glycolic acid) nanofiber mat and an alginate-gelatin hydrogel for wound healing. J. Mater. Chem. B.

[214] Han Q., He J., Bai L., Huang Y., Chen B., Li Z., Xu M., Liu Q., Wang S., Wen N. (2024). Injectable bioadhesive photocrosslinkable hydrogels with sustained release of kartogenin to promote chondrogenic differentiation and partial-thickness cartilage defects repair. Adv. Healthc. Mater..

[215] Mai B., Jia M., Liu S., Sheng Z., Li M., Gao Y., Wang X., Liu Q., Wang P. (2020). Smart hydrogel-based dvdms/bfgf nanohybrids for antibacterial phototherapy with multiple damaging sites and accelerated wound healing. ACS Appl. Mater. Interfaces.

[216] Gheorghiță D., Moldovan H., Robu A., Bița A.I., Grosu E., Antoniac A., Corneschi I., Antoniac I., Bodog A.D., Băcilă C.I. (2023). Chitosan-Based Biomaterials for Hemostatic Applications: A Review of Recent Advances. Int. J. Mol. Sci..

[217] Doppalapudi S., Jain A., Khan W., Domb A.J. (2014). Biodegradable polymers-an overview. Polym. Adv. Technol..

[218] Moss J.A., Mark S.S., Hixon S., Ashley F.T., Chang J.Y., Janda K.D. (2006). Solid-phase synthesis and kinetic characterization of fluorogenic enzyme-degradable hydrogel cross-linkers. Biomacromolecules.

